# A classification‐occupancy model based on automatically identified species data

**DOI:** 10.1002/ecy.70086

**Published:** 2025-05-07

**Authors:** Ryo Ogawa, Frédéric Gosselin, Kevin F. A. Darras, Stephanie Roilo, Anna F. Cord

**Affiliations:** ^1^ Agro‐Ecological Modeling Group Institute of Crop Science and Resource Conservation, University of Bonn Bonn Germany; ^2^ Chair of Computational Landscape Ecology TUD Dresden University of Technology Dresden Germany; ^3^ INRAE, UR EFNO Nogent‐sur‐Vernisson France

**Keywords:** automated biodiversity monitoring, Bayesian statistics, BirdNET, false positive, model integration, passive acoustic monitoring, probabilistic sampling, species classifier

## Abstract

Occupancy models estimate a species' occupancy probability while accounting for imperfect detection, but often overlook the issue of false‐positive detections. This problem of false positives has gained attention recently with the rapid advancement of automated species detection tools using artificial intelligence (AI), which generate continuous confidence scores for each species detection. Novel occupancy models have been introduced that integrate these confidence scores to identify false positives, but these models require thorough assessments of diagnosis and validation. Here, we propose a new occupancy model based solely on AI‐detected species data. We conducted simulations to examine the inferential and predictive accuracies with known true parameters and analyzed AI‐detected species data to test the practical usefulness through goodness‐of‐fit tests and evaluation with external data. Our proposed model mostly outperformed alternative models that ignore imperfect detection or false‐positive error probabilities in terms of accuracy in simulation analyses and goodness‐of‐fit tests in the case study, but not in terms of discrimination metrics based on external data. The proposed occupancy model aids in understanding species–habitat relationships and developing automated biodiversity monitoring workflows by accounting for both false‐negative and false‐positive errors.

## INTRODUCTION

The field of occupancy models, which estimate a species' occupancy probability while accounting for imperfect detection (MacKenzie et al., 2002)—that is, false negatives—has been transformed by technological progress. Traditional methods rely on labor‐intensive human surveys, resulting in limited data collection. Modern techniques combined with artificial intelligence (AI), in contrast, allow for extensive and standardized data collection with less human effort. These include environmental DNA (eDNA) sampling, which uses polymerase chain reaction (PCR) to detect target species presence (Serrao et al., 2018); camera trapping, combined with automated image processing identifying species from captured images (Lonsinger et al., 2024); and passive acoustic monitoring (PAM) coupled with audio recognition models identifying sound‐producing animals (Wood et al., 2024). These advancements have deepened our understanding of species–habitat relationships and can help us make a significant step toward automated biodiversity monitoring (Besson et al., 2022).

The advent of automated species detection tools (hereafter called species classifiers) has also revealed a challenge previously overlooked in occupancy models: false positives (i.e., species misidentifications) can lead to an overestimation of species' occupancy estimates, which in turn can cause inaccurate variable inference. While occupancy models accounting for false positives have been developed previously (Chambert, Grant, et al., 2018; Chambert, Waddle, et al., 2018; Miller et al., 2011; Royle & Link, 2006), the development has been limited by the lack of reliable scores to quantify the chance of potential false positives. Today, species classifiers provide continuous confidence scores for each detection (Aodha et al., 2022; Kahl et al., 2021), with higher scores indicating a greater chance of true positives.

To mitigate false positives in occupancy models, deterministic approaches have been applied, such as the use of threshold cutoffs along the continuous confidence score axis (Singer et al., 2024). This simple approach can reduce the bias caused by false positives and has been applied to occupancy models in eDNA sampling (Serrao et al., 2018), camera trap image recognition (Clare et al., 2019), and sound recognition models (Wood et al., 2024; Wood & Kahl, 2024). However, increasing cutoff thresholds not only gradually removes false positives but also increasingly excludes true positives. Consequently, threshold cutoffs at the optimal point do not guarantee the complete removal of misidentifications while keeping all true detections, due to the unknown true threshold and the non‐probabilistic nature of arbitrary cutoffs.

As an alternative, a probabilistic sampling approach has been recently introduced in occupancy models (Cole et al., 2022; Kéry & Royle, 2020; Rhinehart et al., 2022). These models have combined the occupancy model with a classification model that probabilistically classifies true and false positives along the continuous confidence score axis (hereafter called classification‐occupancy model). This probabilistic approach may provide more accurate estimates of species presence reflecting their inherent uncertainty, unlike the threshold‐cutoff approach which necessarily contains false positives and excludes true positives. However, the failure of parameter convergence is a problem for these complex models that rely on AI‐detected data (Kéry & Royle, 2020; Rhinehart et al., 2022), with label switching during the classification process being a major issue (Stephens, 2000). Hence, a novel classification‐occupancy model requires thorough assessments of model diagnosis, goodness‐of‐fit test, and external validation.

**FIGURE 1:**
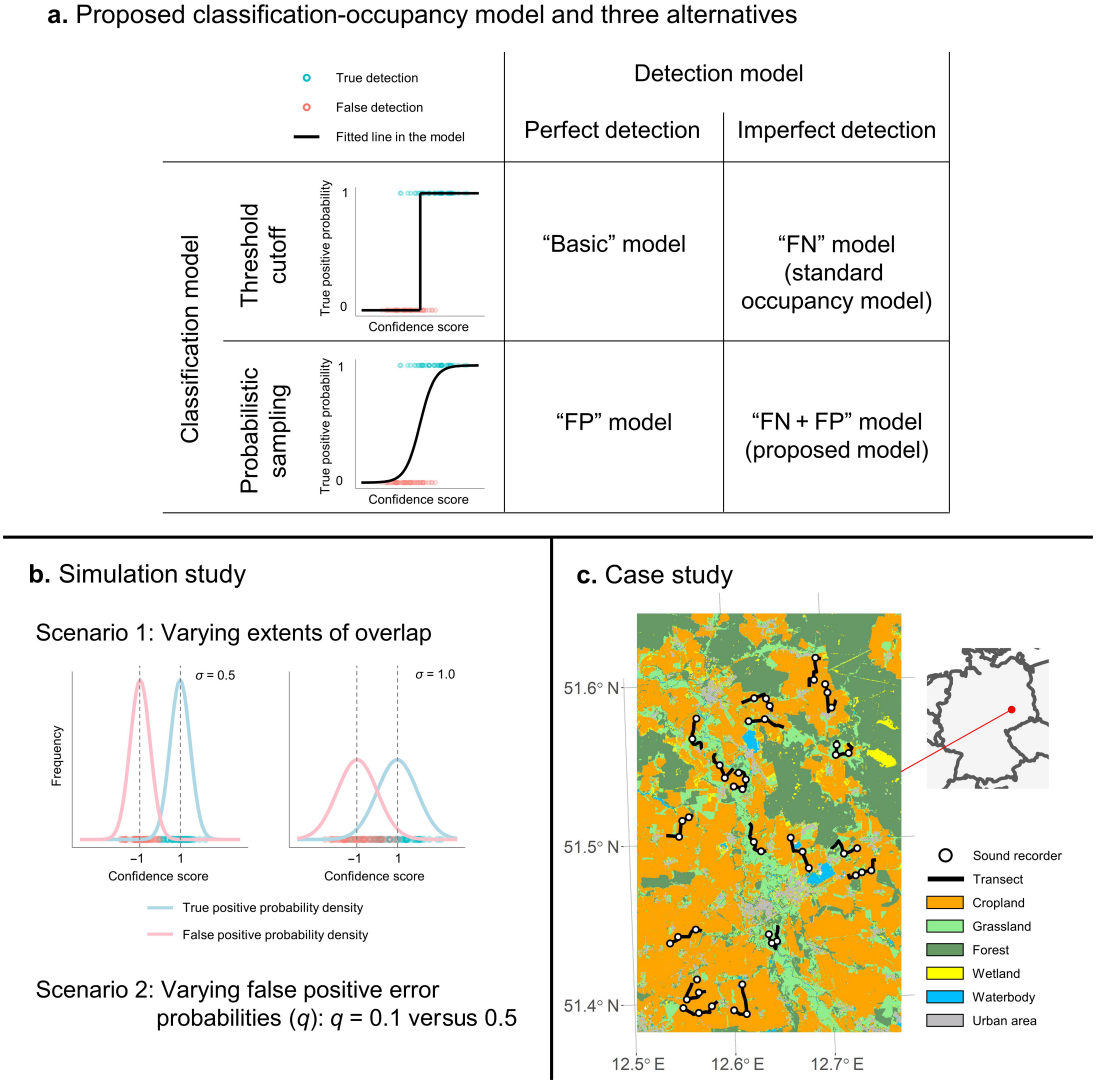
Overview of study workflow to evaluate the classification‐occupancy model. (a) General specification of the proposed classification‐occupancy model (i.e., “FN + FP” model) and three alternative models based on automatically identified species data. “Basic”: model assuming perfect detection and imposing a threshold to reduce false positives; “FN”: model estimating false‐negative detection parameters; and “FP”: model estimating false‐positive detection parameters. (b) Scenarios examined in the simulation study, testing the effect of two scale parameters in normal distributions (Scenario 1) and two false‐positive error probabilities (Scenario 2) on the accuracy of model estimates across four models. (c) Study area of our case study in Saxony, Germany.

We therefore aimed to build a classification‐occupancy model using only AI‐detected species data, ensuring acceptable model convergence, fitting, and predictability. We first propose a Bayesian classification‐occupancy model adapted to AI‐detected species data with continuous confidence scores, considering imperfect detection and probabilistic classification (the “FN + FP” model in Figure 1a). For comparison, we constructed three alternative models as follows (Figure 1a):

1. The “Basic” model assumes perfect detection and imposes a threshold to continuous confidence scores for classification.
2. The “FN” (false negatives) model accounts for imperfect detection and imposes a threshold on continuous confidence scores for classification (i.e., standard occupancy models).
3. The “FP” (false positives) assumes perfect detection and uses probabilistic classification.

We assessed the inferential and predictive accuracies of our proposed model through a simulation study and compared it with the three alternatives (Figure 1b). We then fitted the classification‐occupancy model to real acoustic survey data from a case study in an agricultural landscape (Figure 1c), assessing its goodness‐of‐fit and predictive performance validated by external data. We concluded by discussing its practical applications and limitations based on our findings.

## METHODS

### Modeling framework

#### Standard occupancy model

In the standard occupancy model (the “FN” model in Figure 1a), input data follow the encounter history format based on a capture–recapture modeling framework (Otis et al., 1978): Let *y*_*ij*_ be a species' observation at site *i* and sampling occasion *j* (i.e., the *j*th survey visit of site *i*; Appendix S1: Table S1). If the species is observed, *y*_*ij*_ = 1, and if not observed, *y*_*ij*_ = 0. The model depicts the species' site occupancy probability (ѱ_*i*_) based on an unobservable occupancy status (*z*_*i*_):

(1) $$z_{i}\sim \text{Bernoulli}\left(\psi_{i}\right)$$

Next, the model links the observations with the occupancy status, considering imperfect detection (*p*_*ij*_) at each site and occasion, and assuming no false‐positive detections:

(2) $$y_{\mathrm{ij}}\sim \text{Bernoulli}\left(z_{i}\times p_{\mathrm{ij}}\right)$$

Lastly, the model relates the occupancy probabilities and detection probabilities to environmental predictors:

(3) $$\text{logit}\left(\psi_{i}\right)={X_{\mathrm{occ}}}_{i}\times \beta_{\mathrm{occ}}$$

(4) $$\text{logit}\left(p_{\mathrm{ij}}\right)={X_{\det}}_{\mathrm{ij}}\times \beta_{\det}$$

where ${X_{\mathrm{occ}}}_{i}$ and ${X_{\det}}_{\mathrm{ij}}$ indicate predictor vectors for occupancy probabilities at site *i* and for detection probabilities at site *i* and sampling occasion *j*, respectively. β_occ_ and β_det_ are the coefficient vectors for occupancy and detection, respectively.

#### False‐positive occupancy model

In our false‐positive occupancy model, the species detection can result from three mutually exclusive events:

1. The species is present at the site and detected: *z*_*i*_ × *p*_*ij*_;
2. The species is present at the site, but the signal from the species was not detected. Instead, another signal, not originating from the target species, was detected—corresponding to a false positive: *z*_*i*_ × (1 − *p*_*ij*_) × *w*_*ij*_; or
3. The species is absent from the site, and there is a false positive: (1 − *z*_*i*_) × *w*_*ij*_.

where *w*_*ij*_ denotes a binary process that a detection status is true (*w*_*ij*_ = 0) or false (*w*_*ij*_ = 1) at site *i* and occasion *j*, when *y*_*ij*_ = 1. The summation of these events, the probability of a species detection, is described as follows:

(5) $$z_{i}\times p_{\mathrm{ij}}+z_{i}\times \left(1-p_{\mathrm{ij}}\right)\times w_{\mathrm{ij}}+\left(1-z_{i}\right)\times w_{\mathrm{ij}}=z_{i}\times p_{\mathrm{ij}}+\left(1-z_{i}\times p_{\mathrm{ij}}\right)\times w_{\mathrm{ij}}$$

The second event is absent in the original false‐positive occupancy model proposed by Royle and Link (2006), but we consider that this event can occur in our case study using a species classifier. In summary, the observation model for species detection events is modified as follows:

(6) $$y_{\mathrm{ij}}\sim \text{Bernoulli}\left(z_{i}\times p_{\mathrm{ij}}+\left(1-z_{i}\times p_{\mathrm{ij}}\right)\times w_{\mathrm{ij}}\right)$$

(7) $$w_{\mathrm{ij}}\sim \text{Bernoulli}\left(q_{\mathrm{ij}}\right)\;\left(\text{when}\;y_{\mathrm{ij}}=1\right)$$

where *q*_*ij*_ ~ Uniform(0, 1), representing a false‐positive error probability at site *i* and occasion *j*. Note that we fix *w*_*ij*_ = 0 when *y*_*ij*_ = 0 so that the non‐detection process is explicitly specified as true detection status.

#### Classification model

In the classification model, we link the observed confidence scores to parameters in the false‐positive occupancy model, specifically ѱ_*i*_, *p*_*ij*_, and *q*_*ij*_. Unlike the Gaussian mixture model developed by Kéry and Royle (2020) and Rhinehart et al. (2022), which handle multiple scores per site and occasion, our model uses only one continuous confidence score with each species detection at site *i* and occasion *j* (i.e., *x*_*ij*_). The process of this classification model is conditional only when a species is detected (i.e., *y*_*ij*_ = 1). Our modified approach is a pragmatic reason in the case study to facilitate parameter convergence and reduce computational time and memory usage due to input data inflation caused by species detections every 3 s generated by the species classifier.

We assume the confidence scores for each of the true‐ or false‐positive detections conform to a normal distribution (Assumption 4; Appendix S1: Table S2, but see alternative in Cole et al., 2022)—a widely used statistical distribution that can be flexibly adapted from a 0–1 scale by a logit transformation (e.g., the confidence score in BirdNET; Wood & Kahl, 2024) or from a >0 scale by a log transformation (e.g., the copy number in eDNA sampling; Serrao et al., 2018);

(8) $$x_{\mathrm{ij}}|g_{\mathrm{ij}}=1\sim \text{Normal}\left(\mu_{1},\sigma \right)$$

(9) $$x_{\mathrm{ij}}|g_{\mathrm{ij}}=2\sim \text{Normal}\left(\mu_{2},\sigma \right)$$

where *g*_*ij*_ represents the type of positive detections, either true (*g*_*ij*_ = 1) or false (*g*_*ij*_ = 2); μ_1_ and μ_2_ indicate the mean values of true‐ and false‐positive detection confidence scores, respectively, with the constraint that μ_1_ > μ_2_ (Assumption 5; Appendix S1: Table S2) with a shared scale parameter σ (Assumption 4: Appendix S1; Table S2). We then construct a categorical distribution where every event of true‐ or false‐positive detection at site *i* and occasion *j* is sampled from two probabilities: detection is either true (Pr (*g*_*ij*_ = 1)) or false (Pr (*g*_*ij*_ = 2));

(10) $$g_{\mathrm{ij}}\sim \text{Categorical}\left(\mathrm{Pr}\left(g_{\mathrm{ij}}=1\right),\mathrm{Pr}\left(g_{\mathrm{ij}}=2\right)\right)$$

(11) $$\mathrm{Pr}\left(g_{\mathrm{ij}}=2\right)=1-\mathrm{Pr}\left(g_{\mathrm{ij}}=1\right)$$

#### Model integration

We integrate the false‐positive occupancy model into the classification model by equating the conditional probability of true positives at site *i* and occasion *j*. This is achieved by dividing the true detection probability by the total detection probability.

(12) $$\mathrm{Pr}\left(g_{\mathrm{ij}}=1\right)=\mathrm{Pr}\left(\text{detected signal is true}\right)=\frac{\psi_{i}\times p_{\mathrm{ij}}}{\psi_{i}\times p_{\mathrm{ij}}+\left(1-\psi_{i}\times p_{\mathrm{ij}}\right)\times q_{\mathrm{ij}}}$$

This model integration structure is similar to the one by Cole et al. (2022) and Kéry and Royle (2020), but it is adjusted for the parameters used in our false‐positive occupancy and classification models. Although we use our model integration approach to address practical issues with parameter convergence and input data inflation in our case study, we advise researchers to choose their model integration based on their own study objectives and species classifier used, as long as all conditions of model fitting and computational efficiency are met.

We constructed this proposed model with the Bayesian framework using the software JAGS version 4.3.0 (Plummer, 2003). In both simulation and case study analyses, we set priors of mean confidence scores of true positives: μ_1_ ~ Normal(0, 10); mean confidence scores of false positives μ_2_ ~ Normal(0, 10) with μ_1_ > μ_2_; the scale parameter σ ~ Uniform(0, 10); and both occupancy and detection coefficient parameters: β_occ_ ~ Normal(0, 10) and β_det_ ~ Normal(0, 10).

#### Model assumptions

One main objective of this study was to propose a new occupancy modeling framework (i.e., “FN + FP” model). Therefore, we clarified assumptions related to our proposed model (Appendix S1: Table S2). Besides the assumptions in the standard occupancy model (Assumptions 1–3: Appendix S1: Table S2) and aforementioned Assumptions 4 and 5, we included Assumption 6 for the classification model: Confidence scores exclusively indicate the reliability of true‐positive detections. This assumption might not hold if the scores are affected by factors like the distance between monitoring devices and species locations (Knight & Bayne, 2019; Somervuo et al., 2023). Additionally, sound recognition models can non‐randomly misidentify species due to the presence of a non‐target species with similar vocal characteristics in acoustic surveys (Funosas et al., 2024). This can make it difficult to accurately identify the target species when other non‐target species are vocalizing. Considering these potential assumption violations in our case study, we evaluated whether our proposed classification‐occupancy model showed practical usefulness.

#### Comparison of the proposed model with alternative models

In both simulation and case studies, we compared our proposed model (i.e., the “FN + FP” model) with three alternative models (i.e., the “Basic,” “FN,” and “FP” models; Figure 1a). Classification‐occupancy models (or non‐threshold models; i.e., “FP” and “FN + FP” models) were constructed based on Equations (1) and ((3), (4), (5), (6), (7), (8), (9), (10), (11), (12))–((3), (4), (5), (6), (7), (8), (9), (10), (11), (12)), whereas threshold‐cutoff models (i.e., “Basic” and “FN” models) were constructed based on Equations ((1), (2), (3), (4))–((1), (2), (3), (4)).

For the “Basic” and “FN” models, encounter history input data were generated using a threshold based on continuous scores, which minimized both false‐negative and false‐positive error rates. Scores larger than the threshold were considered as observed, whereas those smaller were considered as not observed. In the simulation study, the threshold was selected based on prior knowledge of μ_1_ and μ_2_ (see detail in *Simulation study*). In the case study, it was determined based on the dataset of expert verification, with corresponding continuous scores generated by the sound recognition model BirdNET (Kahl et al., 2021; see detail in *Case study*). Note that in real data, multiple true and false detections can happen per site and sampling occasion, but our model structure treats any detection, whether single or multiple, as one detection per site and occasion as observed (*y*_*ij*_ = 1).

Both “Basic” and “FP” models assumed perfect detection (i.e., *p* = 1). Although it was rare to assume perfect detection in occupancy models, this comparison allowed us to disentangle the effect of the respective model structures when presenting novel occupancy models: for example, Doser et al. (2023) fitted six different models, two of which assumed perfect detection, when presenting their multi‐species occupancy model. Additional details of these “Basic” and “FP” models are given in Appendix S1: Section S1. The JAGS language scripts of all models and subsequent simulation and case study codes are available in Ogawa (2024).

### Simulation study

In our simulation study, we aimed to assess the inherent behavior of our proposed model and to contrast it with alternative models by using known true parameters (Saas & Gosselin, 2014). When generating simulation data, we fixed the mean confidence scores for true positives, μ_1_ = 1, and false positives, μ_2_ = −1. This allowed us to determine the optimal threshold point for the “Basic” and “FN” models in advance: Detection *y*_*ij*_ = 1 when $x_{\mathrm{ij}}>\frac{\left(\mu_{1}+\mu_{2}\right)}{2}=0$, and non‐detection *y*_*ij*_ = 0 when *x*_*ij*_ ≤ 0.

We assigned two occupancy coefficients (i.e., intercept and one predictor) and one detection coefficient (i.e., intercept only), both randomly selected from a uniform distribution ranging between −1 and 1. The numbers of sites and occasions were set to 50 and 20, respectively, to align with site and occasion numbers in our case study.

We tested two scenarios to understand how accurately the four models predicted the estimates of the true parameter values (Figure 1b). First, we assigned the scale parameter (σ) of the normal distribution to be a value of either 0.5 or 1.0. We expected that the “FN + FP” model would perform better than the “FN” model (i.e., standard occupancy model) when there was greater mixing of true‐ and false‐positive distributions along the confidence score axis. This was because the threshold‐cutoff approach could not efficiently separate true‐ and false‐positive detections when the overlap between the two distributions was large. Second, we set the false‐positive error probability (i.e., *q*) at either 0.1 or 0.5. We expected that the “FN + FP” model would more accurately predict the true parameter values when the false‐positive probability was higher (i.e., *q* = 0.5) than when the false‐positive probability was lower (i.e., *q* = 0.1). This was because a lower occurrence of false positives could lead to data scarcity in false‐positive detections when distinguishing between true and false positives in the classification model. Based on these parameter settings, we generated 100 simulated data points for each combination of σ and *q*.

In our simulation study, we evaluated the success rate of parameter convergence across four models (Figure 1a) under equal conditions. This allowed us to fairly assess how likely each model reached convergence with different model complexities. Therefore, we ran three chains, each with 5000 burn‐in iterations, 50,000 posterior samples after burn‐in, and a thinning rate of one, resulting in a total of 150,000 Markov chain Monte Carlo (MCMC) posterior samples for each simulation. We examined parameter convergence using Gelman–Rubin statistics R‐hat (Gelman et al., 2014). If we found R‐hat >1.1 for at least one parameter in a simulation, we considered the model a failure and calculated the success rate of model convergence (in percent). Low numbers of effective sample sizes also produce unreliable parameter estimates and 95% credible interval (CRI). Therefore, given the models that showed R‐hat <1.1 for all parameters (i.e., successfully converged models), we extracted the lowest effective sample size among all monitored parameters per simulation and calculated the mean of the values over the simulations of successfully converged models. To assess the accuracy of parameter estimation, we evaluated the 95% CRI coverage rates of occupancy probabilities (ψ: a vector of occupancy probabilities ψ_*i*_) and coefficients (β_occ_) (i.e., how often 95% CRIs of estimates overlapped with true parameters). We lastly calculated the mean of the root mean square errors (RMSEs) between the true and estimated ψ and β_occ_ as another quantitative accuracy measure.

### Case study

#### Model species and region

We evaluated the practical usefulness of the classification‐occupancy model in real data analysis by predicting the occupancy of five farmland bird species, namely Eurasian skylark (*Alauda arvensis*), common whitethroat (*Curruca communis*), yellowhammer (*Emberiza citrinella*), Eurasian tree sparrow (*Passer montanus*), and western yellow wagtail (*Motacilla flava*) in Saxony, Germany (Figure 1c). These species were the most frequently observed in line‐transect surveys conducted by humans, providing a sufficient sample size of external test data for our case study (Appendix S1: Table S3). The study area is a predominantly agricultural region with a total area of 285.3 km^2^, comprising 49.2% cultivated crops, 15.7% agricultural grassland, 26.2% forest, 4.3% urban areas, 1.5% water bodies, and 2.0% wetland (Malinowski et al., 2020).

#### Data collection and preprocessing

Line‐transect bird surveys were conducted by ornithologists at 18 locations (Figure 1c) during two periods: the first from May 16 to 19, 2022, and the second from June 5 to 9, 2022. During the transect surveys, 49 automated sound recorders (AudioMoth 1.1 and 1.2, Hill et al., 2019) were deployed along the transects (Figure 1c). We analyzed bird vocalization data in recordings collected between 05:30 AM and 11:30 AM in the period from May 23 to 25. We split the survey period into 1‐h time bins as the unit of sampling occasion *j*. Thus, the total numbers of sampling sites and hours of recordings were 49 and 18 (3 days × 6 h‐time‐bins), respectively. The minimum distance between automated recorders was 383 m, which is greater than the estimated sound extinction distance among varying frequencies and microphone directions, determined by our field calibration (i.e., 160 m; see details in Appendix S1: Section S3 and Table S4; Darras et al., 2016). Therefore, we assumed the distance between the sampling sites to be large enough to avoid spatial autocorrelation of site occupancy probabilities.

We used the deep‐learning convolutional neural network (CNN) BirdNET Analyzer version 2.2 to automatically identify bird vocalizations in sound recordings (Kahl et al., 2021). All BirdNET settings were kept at their default values for species detection (e.g., minimum confidence score = 0.1 and overlap of prediction segments = 0.0). We used the centroid coordinates of the region (i.e., 51.5° N, 12.6° E) as input. BirdNET annotated each 3‐s snippet with species names and their confidence scores.

For “Basic” and “FN” models, annotations were aurally and visually verified on spectrograms by the same ornithologists who conducted transect surveys using the review mode of an online software tool (ecoSound‐web, Darras et al., 2024). The resulting classified annotations were further grouped into true‐positive or false‐positive detections (see details in Appendix S1: Section S2). Using these verified annotations with their associated confidence scores, we determined the optimal threshold per species, which is defined as the point that maximizes the sum of the true‐positive rate (sensitivity) and the true‐negative rate (specificity).

For “FP” and “FN + FP” models, we selected the detection with the highest confidence score within each 1‐h sampling interval, in cases where multiple detections were recorded. Note that we did not use any verified annotations to build the non‐threshold “FP” and “FN + FP” models, where the optimal threshold is priorly unknown. We transformed the BirdNET confidence score using a logit function (Wood & Kahl, 2024). We then fitted the transformed score into the classification model.

We examined the effect of seven land cover types (Malinowski et al., 2020, based on Sentinel‐2 remote sensing data, year 2017) as well as small woody features (European Environment Agency, 2023, years 2018–2021; see details in Appendix S1: Section S4) on the occupancy probability. Land cover information was extracted within a 160‐m radius around the locations of the sound recorders, a radius determined through our field calibration method (Appendix S1: Section S3; Darras et al., 2016). To avoid multicollinearity for these variables, we conducted a principal components analysis on these variables (Zhang et al., 2023) and selected the first three components as predictors of the occupancy probabilities. Lastly, to account for wind sound noises that could reduce the chance of species detection by BirdNET, we modeled the detection probability as a function of hourly wind speed data, downloaded from the fifth generation European Centre for Medium‐Range Weather Forecast (ECMWF) reanalysis for global climate and weather (Hersbach et al., 2023).

#### Model diagnosis

In Bayesian occupancy models, the goodness‐of‐fit is tested by comparing the observed detection data with the data that the model replicated. This comparison is conducted using a method called posterior predictive checks (Gelman et al., 1996; MacKenzie et al., 2018). We summed species detections over hours of recording for each site and calculated the chi‐square discrepancy measure for occupancy models (Kéry & Royle, 2016). For the “FP” and “FN + FP” models, we also calculated the sum of square residuals for classification models. We considered the posterior predictive *p*‐value (PPP) < 0.05 or PPP > 0.95 as a lack of fit.

#### Model evaluation

Using a species classifier (e.g., BirdNET) for both training and testing datasets could inflate the model's predictive performance if the species classifier incorrectly identifies non‐target species as the target during both model building and evaluation processes in a similar manner (i.e., the violation of Assumption 6 in Appendix S1: Table S2; Grimm et al., 2015). To avoid such issues, we used species data collected through line‐transect surveys conducted by humans to evaluate the models, where spatial and temporal coverages are matched to the locations of deployed sound recorders (Figure 1c). Line‐transect data may not be the best dataset for testing model performance due to the potential inflation of pseudo‐absences in the testing data. However, no dataset in bird surveys can provide true absence information, making it difficult to fully validate occupancy probability in the case study. Therefore, we considered this external data just to identify poorly predictive models, rather than to indicate how well the model predicted.

Since line‐transect surveys are different sampling schemes from PAM, they offer complete independence in sampling methods and data characteristics, which can lead to a bias toward more conservative predictive performance rather than optimistic results. Acceptable predictive performance under such conditions can reflect its ability to generalize to new data. We believe this conservative approach is crucial when evaluating the usefulness of novel models: whether a model trained with data from one sampling scheme can predict the pattern of testing data from another sampling scheme.

In this process, we compared predicted species' presence and pseudo‐absence sampled from line‐transect survey data (see details in Appendix S1: Section S5) and calculated three discrimination metrics: the area under the receiver operating characteristic curve (AUC), true skill statistic (TSS), and Sørensen's similarity index (F‐measure). AUC, a threshold‐independent value, ranges from 0 to 1 (Shabani et al., 2018) and indicates a rank‐based measure of binary classification accuracy. TSS ranges from −1 to 1 and is calculated by summing the true‐positive rate (sensitivity) and the true‐negative rate (specificity) and then subtracting one (Allouche et al., 2006). We used the maximum value of the sum of sensitivity and specificity. F‐measure balances the accuracy of positive predictions (precision) and sensitivity (i.e., recall), with scores ranging from 0 to 1 (Sørensen, 1948). We selected the maximum of the balanced mean of precision and sensitivity. For all these metrics, higher scores indicate better model performance.

To gain an adequate effective sample size (>1000) and parameter convergence (R‐hat < 1.1; Gelman et al., 2014) with optimal numbers of burn‐in (set to a minimum of 5000), posterior samples (set to a minimum to 10,000), and thinning rates (set to a minimum of 1 and a maximum of 1000), we used the R package “runMCMCbtadjust” that automatically determines these optimal MCMC settings based on the desired effective sample size (Gosselin, 2024). We also visually assessed traceplots for β_occ_, β_det_, *q*_*ij*_, μ_1_, μ_2_, and σ to ensure the MCMC parameter convergence.

All analyses were conducted using R version 4.4.0 and the following packages: “dismo,” “dplyr,” “ggplot2,” “runjags,” “lubridate,” “mcmcplots,” “sf,” “stringr,” and “terra” (Curtis, 2018; Denwood, 2016; Grolemund & Wickham, 2011; Hadley, 2016; Hijmans, 2022; Hijmans et al., 2021; Pebesma, 2018; R Development Core Team, 2024; Wickham, 2022; Wickham et al., 2023). We provide a step‐by‐step guide to reproduce the method and results of our case study and to be adapted to other case studies and species classifiers (Appendix S2).

## RESULTS

### Simulation study

Our proposed model (i.e., “FN + FP”) had a success rate of parameter convergence ranging from 70% to 90% (Table 1). The effective sample sizes ranged from 492 to 769, except for the case at σ = 0.5 and *q* = 0.1 (effective sample size = 33,383; Table 1). Although these values were lower than those of three alternative models, the “FN + FP” model achieved 95% CRI coverage rates of over 80% for both ψ and β_occ_, except in the scenario at σ = 0.5 and *q* = 0.1 where they attained 44.83%. In contrast, none of the alternative models exceeded coverage rates greater than 50% for either occupancy probabilities (ψ) or coefficients (β_occ_) for any combinations of σ and *q* (Table 1). The “FN + FP” model had the lowest RMSEs for ψ among all four models, although this was not the case for β_occ_ when σ = 1 (Table 1): the “Basic” model showed the lowest RMSE for β_occ_ among all four models when σ = 1, whereas the “FN + FP” models had the lowest RMSE for β_occ_ when σ = 0.5 (Table 1).

**TABLE 1:** Simulation analysis results for the proposed (“FN + FP”) and alternative (“Basic,” “FN,” and “FP”) models.

Metric	σ	*q*	Basic	FN	FP	FN + FP
Convergence success rate (all R‐hat < 1.1; unit: %)	0.5	0.1	100	99	99	82
	0.5	0.5	100	98	100	70
	1	0.1	100	100	99	90
	1	0.5	100	99	98	82
Lowest effective sample size	0.5	0.1	47,146	18,626	13,051	33,383
	0.5	0.5	47,669	16,387	28,089	769
	1	0.1	46,490	20,864	9998	492
	1	0.5	48,967	20,880	5053	804
95% CRI coverage rate (ψ) (unit: %)	0.5	0.1	2.26	3.64	0.18	**44.83**
	0.5	0.5	1.58	1.06	1.24	**93.20**
	1	0.1	1.76	4.86	0.06	**94.22**
	1	0.5	4.82	0.83	4.04	**84.46**
RMSE (ψ)	0.5	0.1	0.27	0.41	0.50	**0.12**
	0.5	0.5	0.24	0.52	0.25	**0.07**
	1	0.1	0.28	0.40	0.50	**0.18**
	1	0.5	0.23	0.51	0.25	**0.11**
95% CRI coverage rate (β_occ_) (unit: %)	0.5	0.1	21.00	**49.49**	48.48	47.56
	0.5	0.5	19.00	47.96	20.00	**95.71**
	1	0.1	20.50	48.00	48.48	**96.67**
	1	0.5	13.50	50.00	34.69	**96.34**
RMSE (β_occ_)	0.5	0.1	0.93	2.81	10.45	**0.40**
	0.5	0.5	0.82	8.18	0.85	**0.41**
	1	0.1	**0.98**	2.54	10.25	2.09
	1	0.5	**0.74**	8.80	0.93	1.17

*Note*: Data were generated from four scenarios, each combining two scale parameters (σ = 0.5 or 1.0) and two false‐positive error probabilities (*q* = 0.1 or 0.5) in the classification model. The lowest effective sample size and two accuracy metrics were computed only when models converged at R‐hat <1.1. The 95% CRI coverage rate indicate how often 95% CRI of estimates overlapped with true parameters out of converged iterations, while RMSEs from the true value for both occupancy probabilities (ѱ) and coefficients (β_occ_) were averaged over converged iterations. The numbers highlighted in bold in coverage rates and RMSE represent the highest accuracy model at each scenario across four models.

Abbreviations: CRI, credible interval; FN, false negative; FP, false positive; RMSE, root mean square error.

### Case study

**FIGURE 2:**
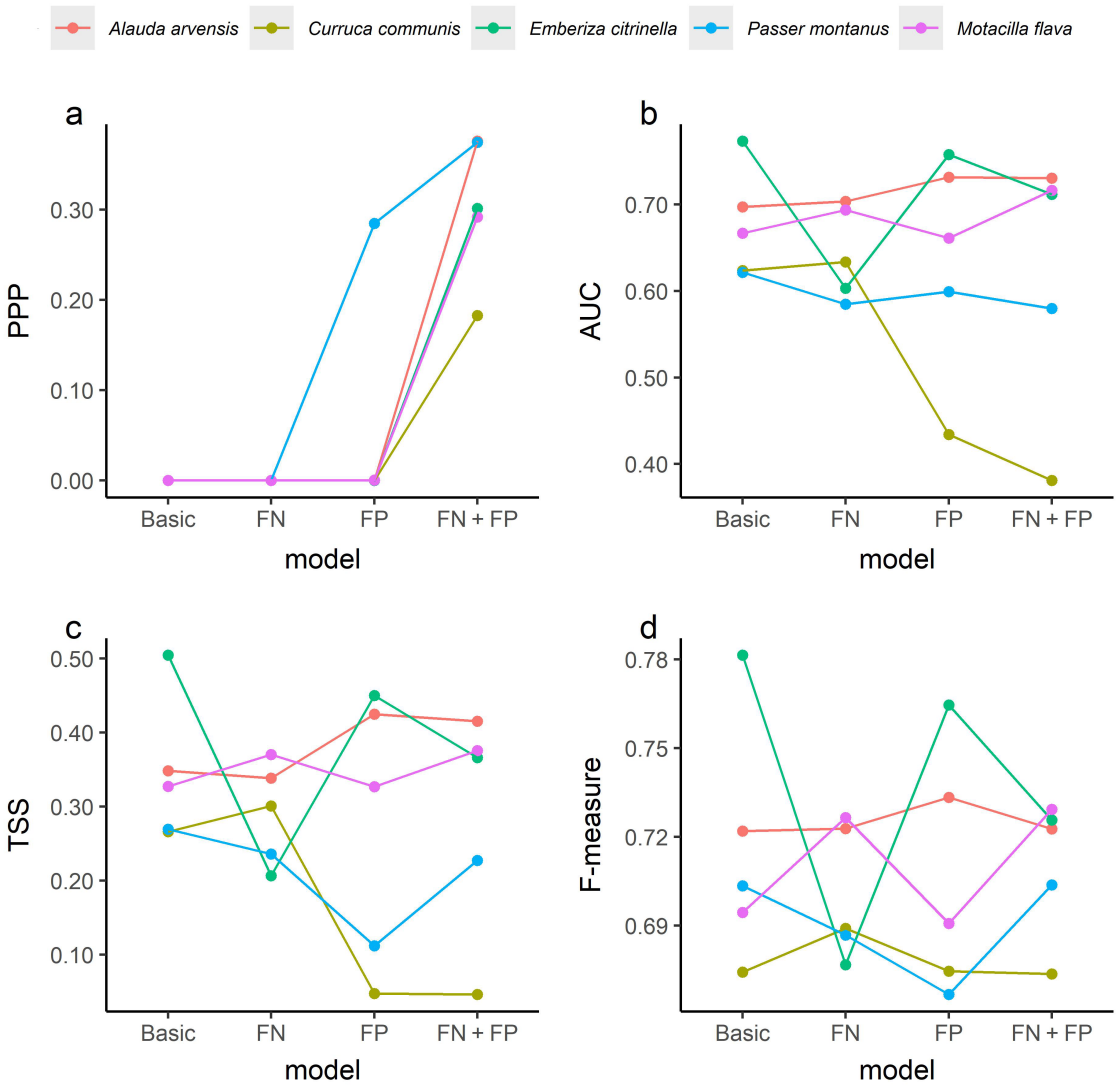
Model diagnosis and evaluation for “Basic,” “FN,” “FP,” and “FN + FP” models on the example of the Eurasian skylark (*Alauda arvensis*), common whitethroat (*Curruca communis*), yellowhammer (*Emberiza citrinella*), Eurasian tree sparrow (*Passer montanus*), and western yellow wagtail (*Motacilla flava*). (a) Posterior predictive *p*‐values (PPP) based on chi‐square discrepancy measure. (b) Area under the receiver operating characteristic curve (AUC), (c) true skill statistic (TSS), and (d) Sørensen's similarity index (F‐measure). Low values of PPP indicate an inconsistency between the model and data based on chi‐square discrepancy, while greater values of AUC, TSS, and F‐measure indicate higher predictive performance of the models.

To achieve adequate effective sample size (>1000) in the “FN + FP” model, we required thinning rates ranging from 666 to 1000 and a total number of posterior samples from 794,731 to 7,663,302 (Appendix S1: Table S5). The “FN + FP” models showed acceptable goodness‐of‐fit for both chi‐square and sum of square discrepancy measures across all species (i.e., 0.05 < PPP < 0.95; Figure 2a and Appendix S1: Figures S1 and S2). Chi‐square discrepancy measures were less than 0.05 for both “Basic” and “FN” models across all species (Figure 2a). The “FP” model showed acceptable goodness‐of‐fit for the Eurasian tree sparrow (i.e., 0.05 < PPP < 0.95), but not for other species (Figure 2a).

**FIGURE 3:**
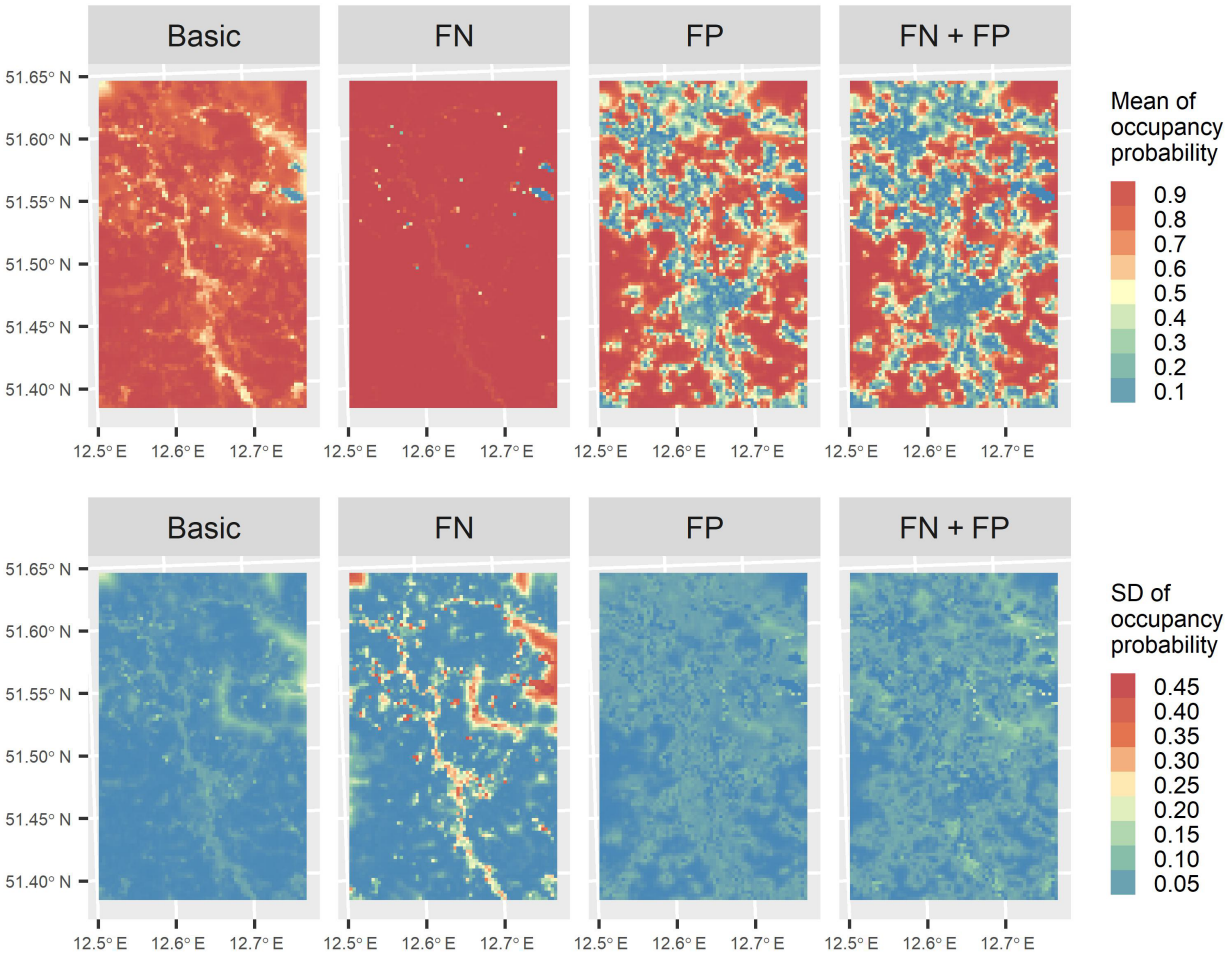
Maps of occupancy probability and its SD for the Eurasian skylark (*Alauda arvensis*) to show model prediction and its uncertainty with “Basic,” “FN,” “FP,” and “FN + FP” models (see Figure 1a for difference). SD is calculated from Markov chain Monte Carlo (MCMC) posterior samples. FN, false negative; FP, false positive.

The “FN + FP” model did not exhibit any noticeable trends of improved predictive performance compared with the alternative models (Figure 2b–d). The “FN + FP” model for the common whitethroat and Eurasian tree sparrow showed lower predictive performance compared with the Eurasian skylark, yellowhammer, and western yellow wagtail (Figure 2b–d). For all species, the predicted species occupancy and their associated uncertainty maps varied largely across the proposed and alternative models (Figure 3 and Appendix S1: Figures S3–S6), where the uncertainty was measured as the SD of species' occupancy probabilities over MCMC posterior samples at each grid cell. For example, the Eurasian skylark distribution maps differed between the “FN + FP” model and the alternative “Basic” and “FN” models (Figure 3). For all models, coefficients of intercept and wind speed in detection models and true‐positive probability along BirdNET confidence score for five species are shown in Appendix S1: Figure S7.

## DISCUSSION

The simulation study highlighted the advantage of the proposed model (i.e., “FN + FP” model) over alternative models, either when there was a large extent of overlap between true‐ and false‐positive detections along the confidence score axis or when the occurrence of false‐positive error probability was high. The case study results demonstrated that (1) alternative models mostly failed to meet the criteria of goodness‐of‐fit and that (2) there were species‐specific cases where classification‐occupancy models showed poor predictive performance even when the goodness‐of‐fit was acceptable. These results highlight the negative consequence of ignoring false negatives and positives in simulation analyses (Table 1). Furthermore, the model fitting result indicated that only the “FN + FP” model was acceptable to interpret estimates in our case study (Figure 2a).

### Simulation study

Our proposed model maintained over 70% of convergence success rates and achieved high 95% CRI coverage rates and low RMSEs for ψ and β_occ_, despite its complexity. In a post hoc analysis, we extended posterior sampling up to 2 days, but not all simulations reached parameter convergence (R‐hat > 1.1). This suggests that certain randomly generated parameter values caused non‐convergence. Conversely, alternative models achieved nearly 100% of convergence success rates but provided inaccurate estimates. This indicates that while our proposed model may struggle with parameter convergence in some cases, it avoids providing inaccurate parameter estimates by checking parameter convergence.

Unexpectedly, the “Basic” model had the lowest RMSE for β_occ_ when the overlap between true‐ and false‐positive detections along the confidence score axis was large at σ = 1.0. However, the 95% CRI coverage rates of the “Basic” model at σ = 1.0 were at most 20.50%, indicating that the 95% CRI coverage rate for β_occ_ was overly precise. RMSE can indicate the degree of deviation between true and estimated parameters, but cannot reflect the direction of bias (Lotterhos et al., 2022). Therefore, RMSE alone may not be the most effective metric for evaluating model accuracy in the comparative analyses among the four models. Instead, considering both 95% CRI coverage rates and RMSE can ensure a more balanced and accurate interpretation of simulation analysis results, both of which were also used in the assessment to introduce a novel occupancy model in the past (e.g., Doser et al., 2023).

### Case study

The case study demonstrated successful parameter convergence (i.e., β_occ_, β_det_, *q*_*ij*_, μ_1_, μ_2_, σ) in the “FN + FP” model of all species. This convergence success can be attributed to the long duration of MCMC posterior sampling, which is a strength of MCMC‐based estimation in hierarchical models when the sample size is low (Rajaratnam & Sparks, 2015). The residual plot of chi‐squared discrepancy measures detected multiple local optima in observed residuals (Appendix S1: Figure S1). The binary latent variables (*z*_*ij*_ and *w*_*ij*_) might have been trapped in local optima during the long posterior sampling without disrupting the MCMC parameter convergence of the monitored priors. Additionally, our method for estimating posterior predictive Bayesian *p*‐value tends to be conservative when accepting goodness‐of‐fit (Conn et al., 2018; Gosselin, 2011). Nevertheless, most alternative models did not meet the criteria of the posterior predictive check given its conservative approach (Figure 2a). This indicates that ignoring false‐negative and false‐positive error probabilities in AI‐detected species data might have caused issues with goodness‐of‐fit in the alternative occupancy models.

Discrimination metrics (i.e., AUC, TSS, and F‐measure) are derived from classification rates, which are based on predicted occupancy probabilities and presence/pseudo‐absence in the testing dataset. Even if the values of the discrimination metrics satisfy a general criterion (e.g., AUC > 0.7 used as a common threshold), these metrics do not necessarily evaluate the absolute values of occupancy probabilities. Instead, they measure the correlations between estimated and actual occupancies (MacKenzie et al., 2018). The value of AUC can also be inflated when pseudo‐absence points are used as absence points (Lotterhos et al., 2022). That may be the reason why predicted species distribution maps of the Eurasian skylark and their associated uncertainties differed between the proposed model and alternatives (Figure 3), while AUC scores were similar across the four models for the Eurasian skylark (Figure 2b). Therefore, we recommend examining goodness‐of‐fit tests before evaluating discrimination metrics.

Rather than emphasizing how well the model fits through discrimination metrics, we placed high importance on the species data collected through line‐transect surveys conducted by humans as an external dataset to eliminate “FN + FP” models which acceptably fitted the data but yielded low predictive performance. For example, the common whitethroat and Eurasian tree sparrow had low predictive performance in our discrimination metrics (Figure 2b–d). For the common whitethroat, this could be because of the violation of Assumption 4, assuming normal distributions in the classification model (see the high frequency of confidence score clustered around 0.9–1.0 in the histogram compared with other species in Appendix S1: Figure S8b). One solution to this problem could be to switch from normal to beta distributions (Cole et al., 2022). Alternatively, using different scale parameters for the Gaussian mixture model might also be considered (Rhinehart et al., 2022).

Detections of the Eurasian tree sparrow might have been mixed up with the similar vocal traits of the house sparrow (*Passer domesticus*), which would violate Assumption 6: The confidence score exclusively indicates the reliability of true species detection. Among our five target species, only the BirdNET scores of the Eurasian tree sparrow had positive relationships with those of the co‐identified house sparrow (Appendix S1: Figure S9d). Indeed, BirdNET can accidentally co‐identify two or more species that have similar vocal characteristics (Funosas et al., 2024). As one analytical solution, one could modify a model structure that addresses species misidentifications (Chambert, Grant, et al., 2018; Chambert, Waddle, et al., 2018). We kept our classification‐occupancy model flexible enough to implement other types of occupancy models (see “Solutions” column in Appendix S1: Table S2) so that experienced model developers can further modify our proposed model structure with the practical guideline documented in Appendix S2.

The next step is to compare our proposed model with a standard occupancy model, with experts examining all snippets at each hour of recording, to evaluate how accurately our model handles false positives. While we only verified a limited number of annotated snippets and could not provide a comparative study, we stress that such an analysis would robustly prove whether our proposed model can infer species–habitat relationships and predict species distributions.

## CONCLUSIONS

We conducted two separate evaluations of the proposed model, using both simulation and case studies. These evaluations provided a more comprehensive assessment of the model's robustness from both a theoretical and practical standpoint. The simulation study highlighted the potential adverse consequence of overlooking false negatives and false positives in occupancy models using AI‐detected species data. The case study results demonstrated that the classification‐occupancy model outperformed alternative models in terms of goodness‐of‐fit, but not discrimination metrics based on external data.

Although further research is needed to address the limitations identified in our case study, our findings underscore the promise of integrating AI approaches with occupancy modeling. The proposed model may be particularly advantageous over the three alternative models tested in cases where accurate ecological inference and predictability are crucial. To ensure the reliability of occupancy maps when applying the proposed model to other case studies, we recommend (1) testing the correlations of confidence scores between co‐identified species and (2) examining the histogram of confidence scores for the normality assumption in the classification model.

## AUTHOR CONTRIBUTIONS

**Ryo Ogawa:** Conceptualization; methodology; software; validation; formal analysis; investigation; data curation; writing—original draft; writing—review and editing; visualization. **Frédéric Gosselin:** Methodology; writing—review and editing. **Kevin F. A. Darras:** Methodology; investigation; writing—review and editing. **Stephanie Roilo:** Investigation; writing—review and editing. **Anna F. Cord:** Conceptualization; resources; project administration; funding acquisition; writing—review and editing.

## CONFLICT OF INTEREST STATEMENT

The authors declare no conflicts of interest.

## Supporting information


Appendix S1.

Appendix S2.

## Data Availability

Data and code (Ogawa, 2024) are available in Zenodo at https://doi.org/10.5281/zenodo.12802535.
