# Supplementary material for: A classification‐occupancy model based on automatically identified species data
Source: Ecology. 2025 May 7;106(5):e70086. doi: 10.1002/ecy.70086 (PMC12056683; doi:10.1002/ecy.70086)
Supplement: Supplementary file 1 — Appendix S1. [file ECY-106-e70086-s001.pdf]

Appendix S1

Journal: Ecology

Title: A classification-occupancy model based on automatically identified species data

Authors: Ryo Ogawa, Frédéric Gosselin, Kevin F. A. Darras, Stephanie Roilo, and Anna F. Cord

Section S1: Short description of the "Basic" and "FP" models

Because we assumed perfect detection (p = 1) in the "Basic" and "FP" models (Figure 1a), the models do not require the distinction of observation and process models. In the "Basic" model, instead of Equations 1 and 2, we replaced the formulas with;

$$y_{ij} \sim Bernoulli(\psi_i)$$

Similarly, in the "FP" model, instead of Equations 1 and 6, we replaced the formulas with;

$$y_{ij} \sim Bernoulli(\psi_i + (1 - \psi_i) \cdot w_{ij})$$

Section S2: Optimal threshold selection through labeling data

Each species' vocalization was first annotated by BirdNET in 3-second snippets and then verified aurally and visually by ornithologists in ecoSound-web's review mode (Darras et al. 2024), categorizing them into "accepted", "corrected", "unclear", or "rejected" identification. We considered "accepted" as true positive detections and "corrected" or "rejected" as false positive detections, and calculated the optimal threshold points for BirdNET scores using expert-verified annotations, aiming to maximize the sum of true positive rates (i.e., sensitivity) and true negative rates (i.e., specificity). We excluded "unclear" data as the uncertain data which cannot be categorized as either a true nor a false positive detection. The maximum sum of specificity and sensitivity was calculated through the "threshold" function in the "dismo" package (Hijmans et al. 2021). Numbers of annotated 3-second snippets were 705 for skylark (*Alauda arvensis*), 627 for common whitethroat (*Curruca communis*), 613 for yellowhammer (*Emberiza citrinella*), 263 for Eurasian tree sparrow (*Passer montanus*), and 582 for western yellow wagtail (*Motacilla flava*).

Section S3: Calibration to determine the range of land cover extraction

We performed a sound detection space measurement to establish the range for extracting land cover variables (i.e., predictors of occupancy probabilities) in our study area. This involved emitting test sounds at known distances to calculate the sound extinction distance, the furthest distance at which sound recorders can detect sound signals of particular frequencies and levels. We selected a subset of 30 audio recording locations and followed the sound detection space calculation methods of Darras et al. (2016). We examined the test sounds of frequencies of 2, 4, 8, and 12 kHz emitted from a loudspeaker at distances of 4, 8, 16, and 32 m, both in front of and behind the recorder.

After the field calibration survey, we extracted the maximum sound levels of each test sound for each site, frequency, direction, and distance. We constructed a linear model for each frequency and direction, with relative sound levels (i.e., amplitude in dB) as the response and log-transformed distance as the explanatory variable. The sound extinction distance was determined by finding the intersection of the linear model's slope and the horizontal line representing the ambient sound level (dB), which was calculated by randomly selecting five audio files from the aforementioned 30 locations. For the first two minutes of each file, we sampled the ambient sound level of four frequencies. We then averaged these amplitudes for each frequency to obtain the ambient sound amplitude. The shortest sound extinction distance among all frequencies was used for our land cover range extraction analysis (i.e., 160 m; Table S4).

Section S4: Environmental data collection and preprocessing

We used seven land cover types. Six of these were sourced from Sentinel 2 land cover data (10 m resolution; Malinowski et al. 2020), and one from small woody structure data (5 m resolution; European Environment Agency 2023). We used these land cover data within a 160 m radius from the sound recording location, a distance determined by sound extinction analysis. We then calculated the proportions of 1) cropland, 2) grassland, and 3) wetland as the herbaceous land cover types of open fields for target species' habitats. Additionally, we calculated the distances to 4) waterbody, 5) urban area, 6) forest covers, and 7) small woody features as the proximity of spatial features within the habitats. To avoid multicollinearity and parameter non-identifiability for these seven variables, we conducted a principal component analysis on these variables (Zhang et al. 2023). We selected the first three components as predictors of occupancy probabilities.

For the detection coefficient, we collected hourly u-winds (i.e., west-to-east winds) and v-winds (i.e., south-to-north winds) at a height of 10 m above the ground at audio recording locations. This data was sourced from the fifth generation European Centre for Medium-Range Weather Forecast (ECMWF) reanalysis for the global climate and weather with a spatial resolution of 0.25 degree (ERA5; Hersbach et al. 2023). We computed the hourly wind speed using the formula: $wind\ speed = \sqrt{(u\text{-}wind)^2 + (v\text{-}wind)^2}$. We then log-transformed the hourly wind speed to meet the normality assumption. The transformed, site- and occasion-specific wind speed was used as the predictor of detection probabilities.

Section S5: Generating pseudo-absence locations by random sampling

Generating pseudo-absence data through a single random sampling event can introduce sampling bias because the single event does not adequately represent the full range of conditions under which other pseudo-absences occur. To avoid the bias, we have implemented the following process that repeats 100 times, reducing the likelihood of bias from a single sampling event;

1. We used species detection locations within a 100-m buffer of each transect line as presence locations.
2. We randomly sampled the pseudo-absence locations, matching the number of presence points for each species. These pseudo-absence locations were selected within a 100 m buffer of transect lines, but also outside a 160 m range of presence points to avoid any spatial overlap between presence and pseudo-absence areas.
3. We extracted the predicted occupancy probability on all presence and pseudo-absence points and calculated three discrimination metrics: area under the curve (AUC), true skill statistics (TSS), and Sørensen's similarity index (F-measure).

Finally, we averaged these discrimination metrics over the 100 times iterations. The average value was used as the resulting discrimination metrics (Figure 2b-d).

Table S1. Description of math symbols used in the classification-occupancy model in the main text.

Symbol type	Math symbol	Description
index	i	survey site ID
	j	sampling occasion ID, indicating the order of a survey event per site
Observables	y_{ij}	Detection (y_{ij} = 1) or non-detection (y_{ij} = 0)
	x_{ij}	Confidence score of species detection, conditional on y_{ij} = 1
	$\boldsymbol{Xocc_i}$	Predictor vector for ψ_i
	$\boldsymbol{Xdet_{ij}}$	Predictor vector for p_{ij}
Unobservables	z_i	Occupancy status: presence (z_i = 1) or absence (z_i = 0)
	w_{ij}	False detection status. When y_{ij} = 1, either false detection (w_{ij} = 1) or true detection (w_{ij} = 0). When y_{ij} = 0, w_{ij} is fixed to 0.
	ψ_i	A species' occupancy probability at site i
	$\boldsymbol{\psi}$	A vector of occupancy probabilities ψ_i
	p_{ij}	True positive detection probability
	q_{ij}	False positive error probability
	g_{ij}	Type of positive detections, either true (g_{ij} = 1) or false (g_{ij} = 2)
	μ_1	Mean of true positive scores in a normal distribution
	μ_2	Mean of false positive scores in a normal distribution
	σ	Scale parameter of true/false positive scores in a normal distribution
	$\boldsymbol{\beta}_{occ}$	Occupancy coefficient vector
	$\boldsymbol{\beta}_{det}$	Detection coefficient vector

Table S2. The assumptions made for our proposed classification-occupancy model.

Model	Assumption	When is assumption violated?	Solutions
False-positive occupancy model	A1. Site occupancy status remains constant during survey periods.	When a species' temporal activity patterns affect their occupancy status per sampling occasion.	Implementation of dynamic occupancy models (Royle and Kéry 2007).
	A2. No dependency in the detection of species between successive samplings at the same site.	When a species is attracted to or avoids traps or monitoring devices after being captured once (so-called trap response, Tanaka 1980).	Implementation of dependency between consecutive sampling occasions in the model or multi-scale occupancy models (Nichols et al. 2008, Hines et al. 2010).
	A3. No spatial autocorrelation between sampling sites.	When species occupancy status is affected by spatial autocorrelation among sites, in addition to environmental factors.	Inclusion of spatial models to mitigate spatial autocorrelation (Saas and Gosselin 2014, Rushing et al. 2019).
Classification model	A4. The confidence scores for both true positive and false positive detections follow normal distributions with a shared scale parameter.	The type of the statistical distribution is not fitted.	Use of other statistical distribution types (Cole et al. 2022).
		The scale parameter is not the same between true and false positive confidence score distributions.	Independent scale parameters for both true and false positive normal distribution (Cole et al. 2022, Rhinehart et al. 2022).
	A5. The mean confidence score of the true positive distribution is always greater than that of the false positive.	Machine learning algorithms generate incomparable confidence scores per species.	Improvement of machine learning algorithms.
	A6. The confidence scores exclusively indicate the reliability of true species detection.	When scores are explained by other factors, for example by the distance between a signal sender and receiver (Knight and Bayne 2019, Somervuo et al. 2023).	Invention of another model framework that implements distance to confidence scores.

		Non-random species misidentification due to the presence of a non-target species with similar vocal characteristics in acoustic surveys.	Preliminary test of correlations of confidence scores between target species and co-identified other species, to examine if the proposed model is suited for the target species. Otherwise, implementing an occupancy model that account for species misidentification (Chambert et al., 2016 & 2018)

Table S3. Species counts used as external test dataset, based on human-based line transect surveys observed within a 100-m buffer area.

Target species	May 16th to 19th	June 5th to 9th	Total
Eurasian skylark (*Alauda arvensis*)	164	139	303
Common whitethroat (*Curruca communis*)	103	79	182
Yellowhammer (*Emberiza citrinella*)	72	90	162
Eurasian tree sparrow (*Passer montanus*)	52	47	99
Western yellow wagtail (*Motacilla flava*)	52	42	94

Table S4. Estimated sound extinction distances for each frequency and sound direction based on the field calibration process.

Source direction	Frequency (kHz)	Extinction distance (m)
front	2	419
**back**	**2**	**159**
front	4	936
back	4	345
front	8	1,737
back	8	399
front	12	504
back	12	198

Table S5. Numbers of burn-in, thinning rate, and posterior sampling for four models (Figure 1a) with five target species. Burn-in iterations were set to a minimum of 5,000; thinning rates were set to a minimum of 1 and a maximum of 1,000; and posterior samples were set to a minimum of 10,000.

Species	Model	Burn-in	Thinning rates	Posterior samples
Eurasian skylark (*Alauda arvensis*)	Basic	5,000	1	10,000
	FN	5,000	32	19,280
	FP	5,000	22	15,830
	FN+FP	5,000	666	1,672,637
Common whitethroat (*Curruca communis*)	Basic	5,000	1	10,000
	FN	5,000	39	24,469
	FP	120,847	1	159,465
	FN+FP	5,000	1,000	3,593,598
Yellowhammer (*Emberiza citrinella*)	Basic	5,000	1	10,000
	FN	5,000	70	41,885
	FP	5,000	1	10,000
	FN+FP	5,000	876	794,731
Eurasian tree sparrow (*Passer montanus*)	Basic	5,000	1	10,000
	FN	5,000	1	10,000
	FP	5,000	596	732,303
	FN+FP	5,000	986	7,663,302
Western yellow wagtail (*Motacilla flava*)	Basic	5,000	1	10,000
	FN	5,000	31	18,990
	FP	5,000	528	294,560
	FN+FP	5,000	875	2,638,557

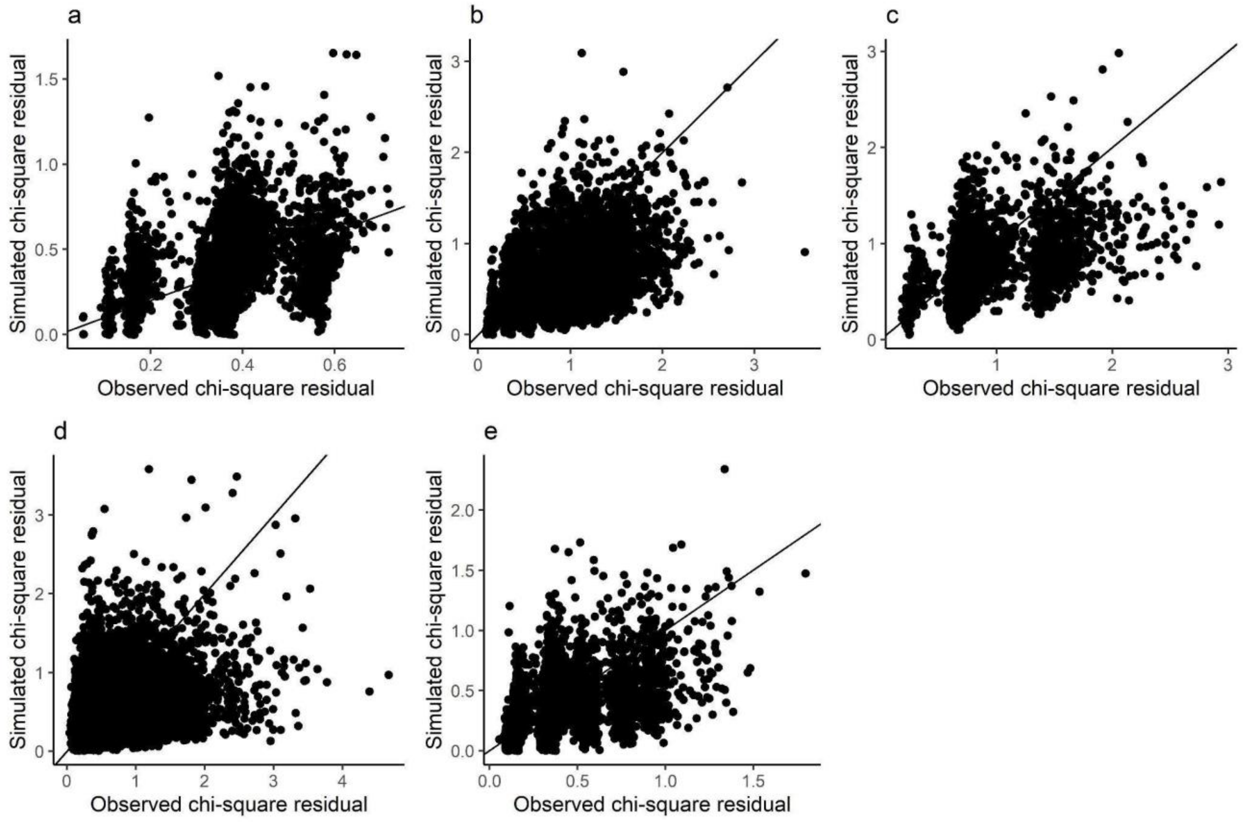

Figure S1. Posterior predictive checks of chi-square discrepancy measures for classification-occupancy model (“FN+FP”). X- & y-axes indicate, respectively, observed and simulated chi-square residuals. Black points represent the MCMC posterior samples and a line in each plot shows y=x to examine whether the points of posterior samples are equally distributed for upper left and lower right sides. **a**. Eurasian skylark (*Alauda arvensis*), **b**. common whitethroat (*Curruca communis*), **c**. yellowhammer (*Emberiza citrinella*), **d**. Eurasian tree sparrow (*Passer montanus*), and **e**. Western yellow wagtail (*Motacilla flava*).

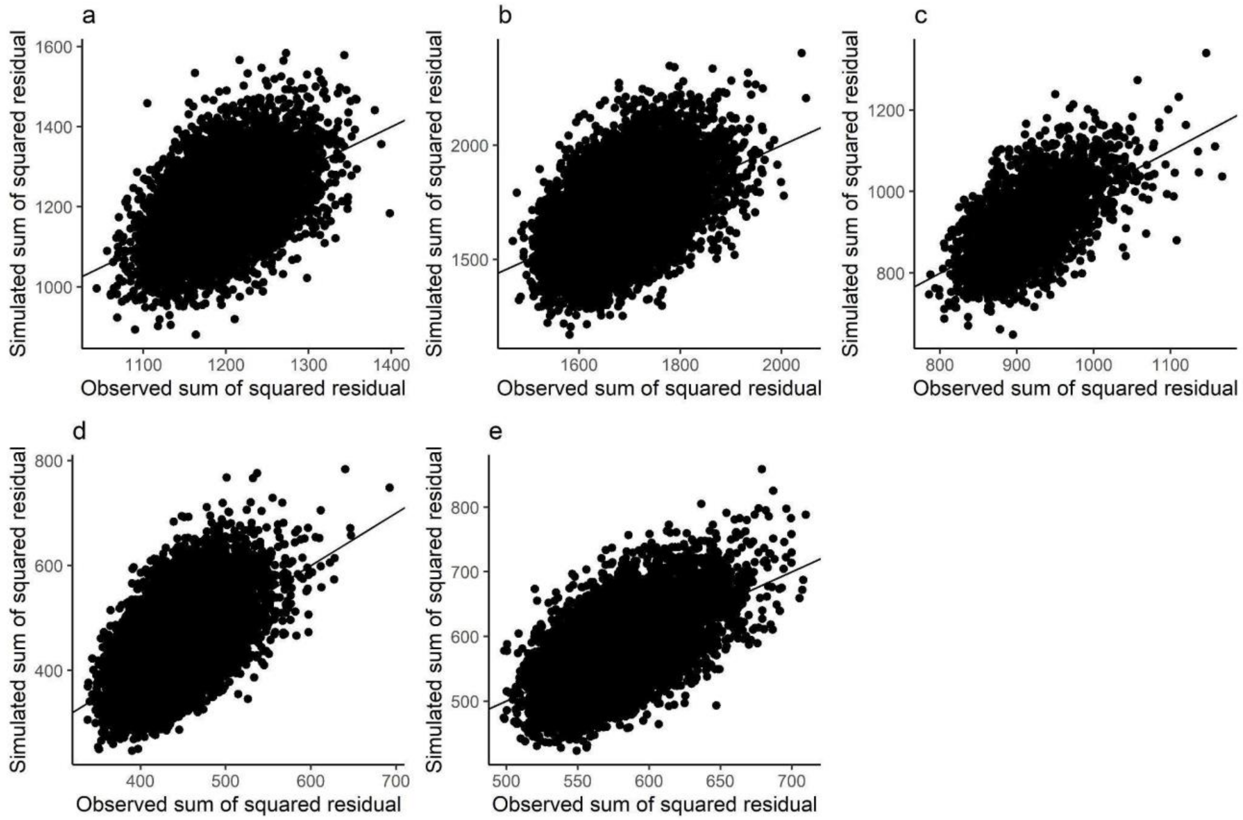

Figure S2. Posterior predictive checks of sum of square discrepancy measures for classification-occupancy model ("FN+FP"). X- & y-axes indicate, respectively, observed and simulated sum of square residuals. Black points represent the MCMC posterior samples and a line in each plot shows y=x to examine whether the points of posterior samples are equally distributed for upper left and lower right sides. **a**. Eurasian skylark (*Alauda arvensis*), **b**. common whitethroat (*Curruca communis*), **c**. yellowhammer (*Emberiza citrinella*), **d**. Eurasian tree sparrow (*Passer montanus*), and **e**. Western yellow wagtail (*Motacilla flava*).

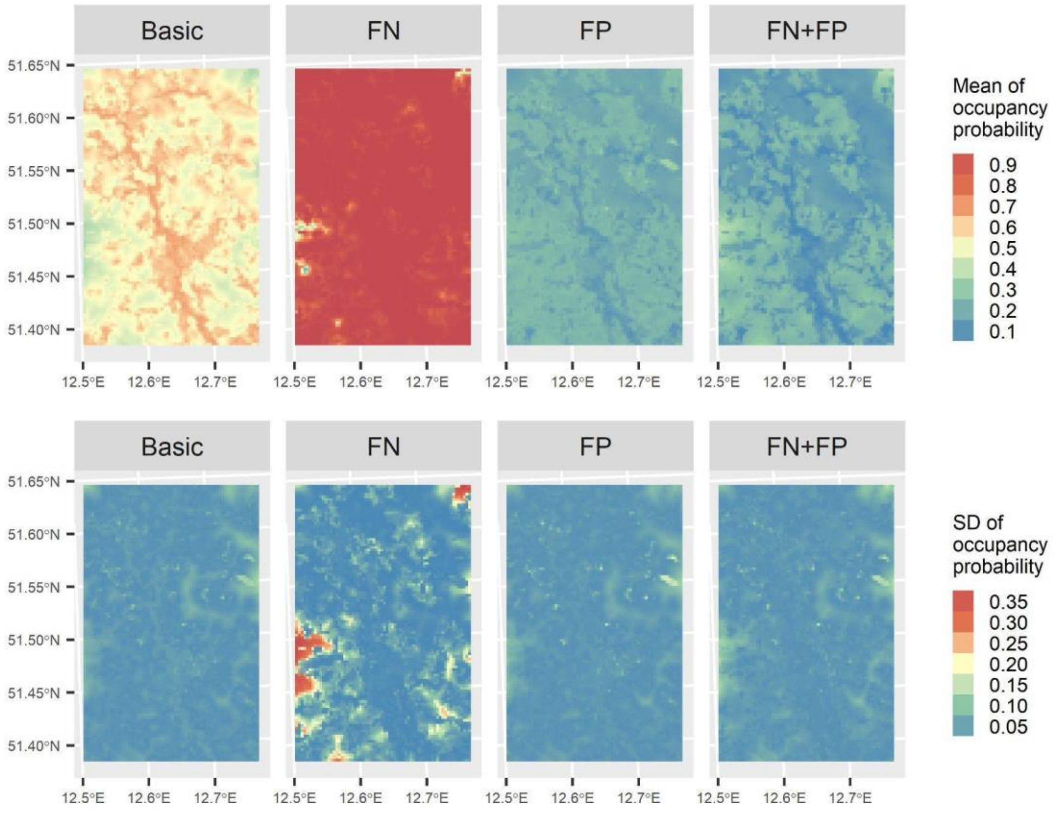

Figure S3. Maps of occupancy probability and its standard deviation (SD) for the common whitethroat (*Curruca communis*) to show model prediction and its uncertainty with “Basic”, “FN”, “FP”, and “FN+FP” models (see Figure 1a for difference). SD is calculated by MCMC posterior samples.

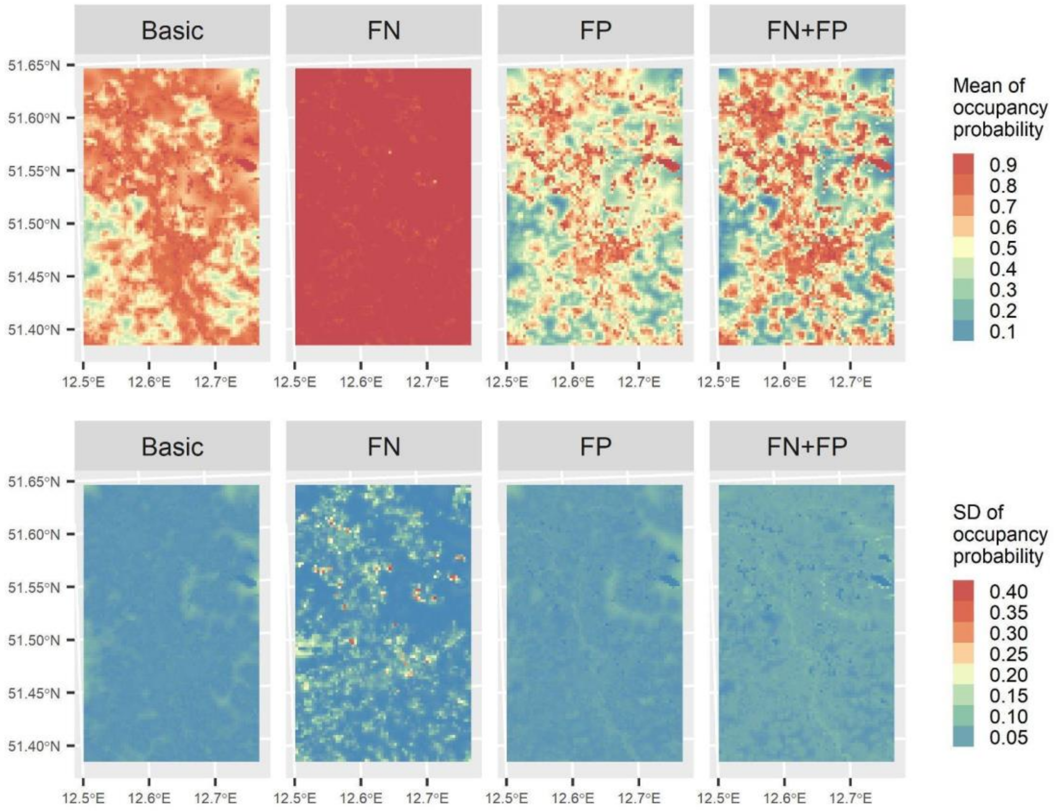

Figure S4. Maps of occupancy probability and its standard deviation (SD) for the yellowhammer (*Emberiza citrinella*) to show model prediction and its uncertainty with “Basic”, “FN”, “FP”, and “FN+FP” models (see Figure 1a for difference). SD is calculated by MCMC posterior samples.

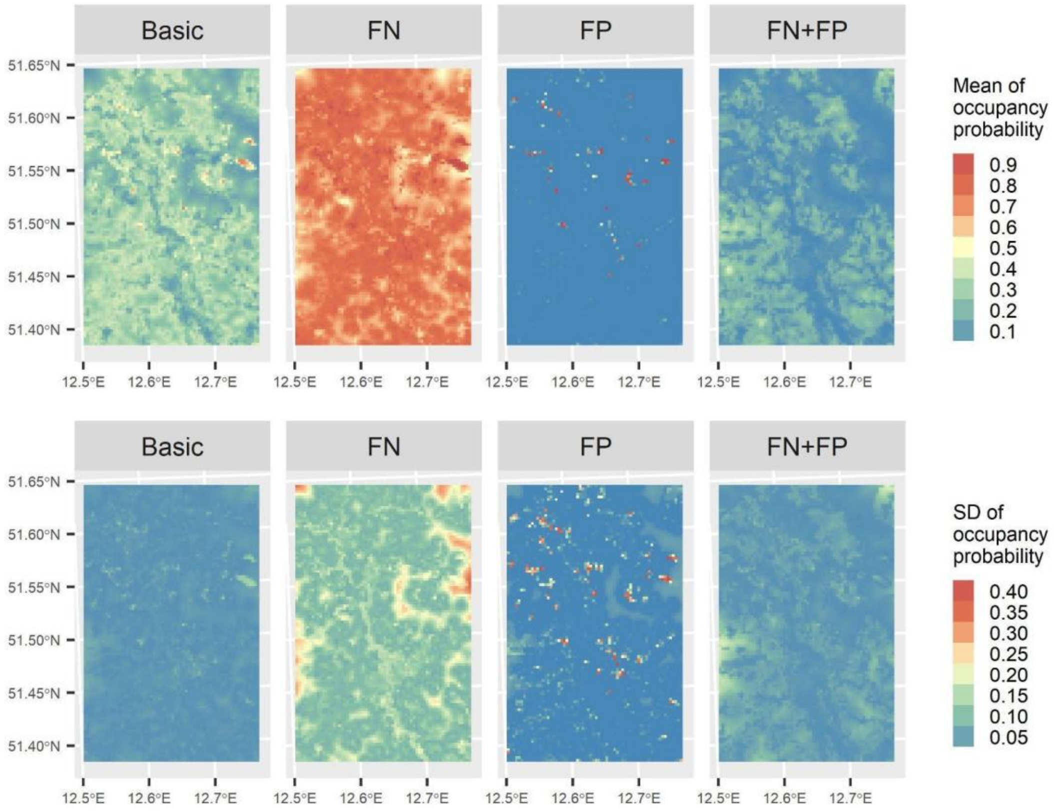

Figure S5. Maps of occupancy probability and its standard deviation (SD) for the Eurasian tree sparrow (*Passer montanus*) to show model prediction and its uncertainty with “Basic”, “FN”, “FP”, and “FN+FP” models (see Figure 1a for difference). SD is calculated by MCMC posterior samples.

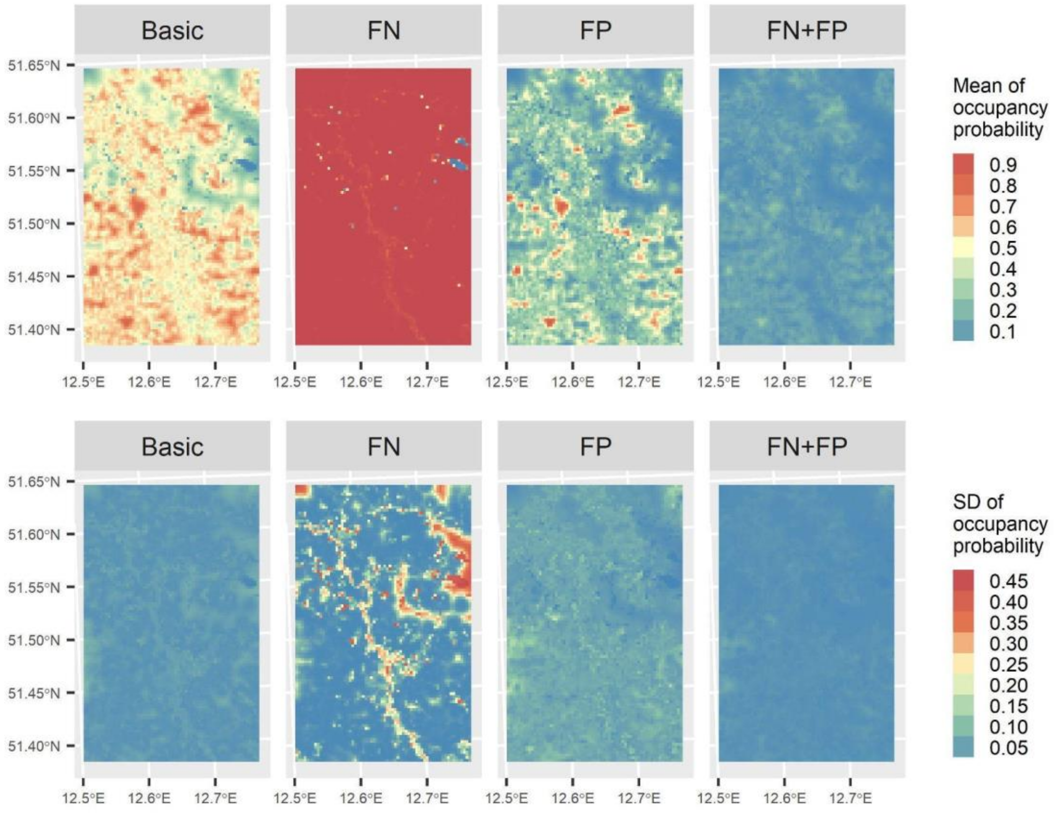

Figure S6. Maps of occupancy probability and standard deviation (SD) for the western yellow wagtail (*Motacilla flava*) to show model prediction and its uncertainty with “Basic”, “FN”, “FP”, and “FN+FP” models (see Figure 1a for difference). SD is calculated by MCMC posterior samples.

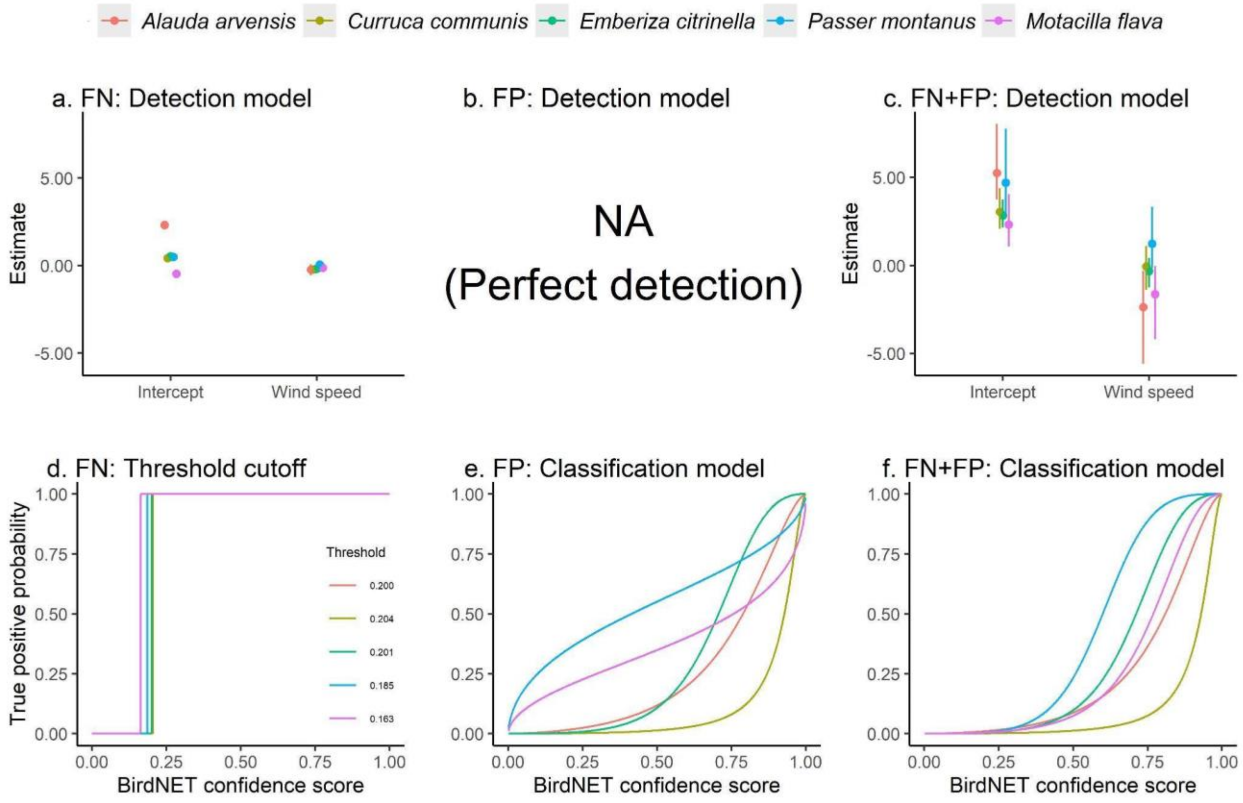

Figure S7. Coefficients of intercept and wind speed in detection models and true positive probability along BirdNET confidence score for five species. **a**. Coefficients of the detection model in the "FN" model. **b**. Perfect detection (therefore NA) in the "FP" model. **c**. Coefficients of the detection model in the "FN+FP" model. **d**. Classification of true and false positive detections by threshold-cutoff approach imposed by 3-second BirdNET expert-verified-annotation. **e**. Relationship curve between the highest BirdNET confidence score within one hour sampling interval and true positive detection probability in the "FP" model. **f**. Relationship curve between the highest BirdNET confidence score within one hour sampling interval and true positive detection probability in the "FN+FP" model. The "Basic" model is not presented because it assumes perfect detection (same as **b**), and because false positive classification is conducted through threshold cutoff (same as **d**).

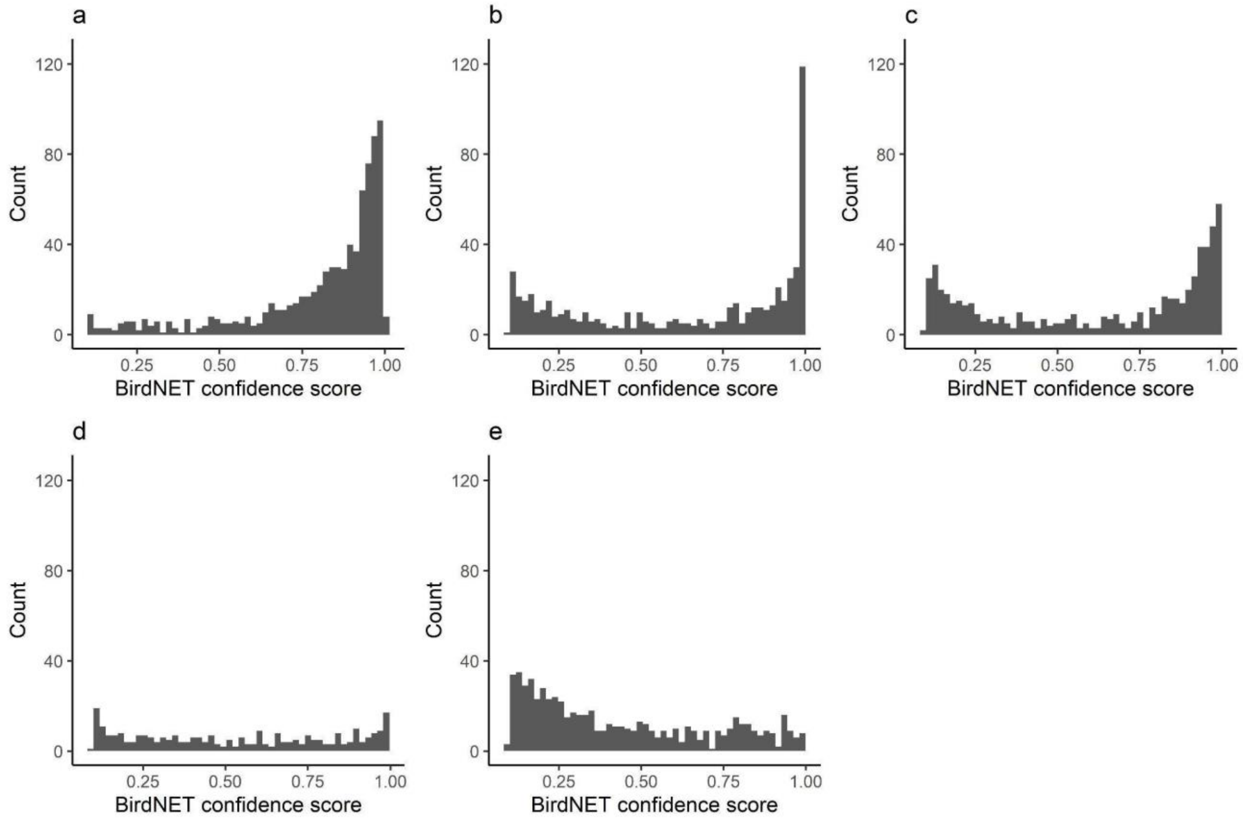

Figure S8. Histograms of BirdNET confidence scores used for classification-occupancy models for five species. X- & y-axes represent, respectively, BirdNET confidence score and the count of detection at each bin of the score. **a**. Eurasian skylark (*Alauda arvensis*). **b**. common whitethroat (*Curruca communis*), **c**. yellowhammer (*Emberiza citrinella*), **d**. Eurasian tree sparrow (*Passer montanus*), and **e**. Western yellow wagtail (*Motacilla flava*).

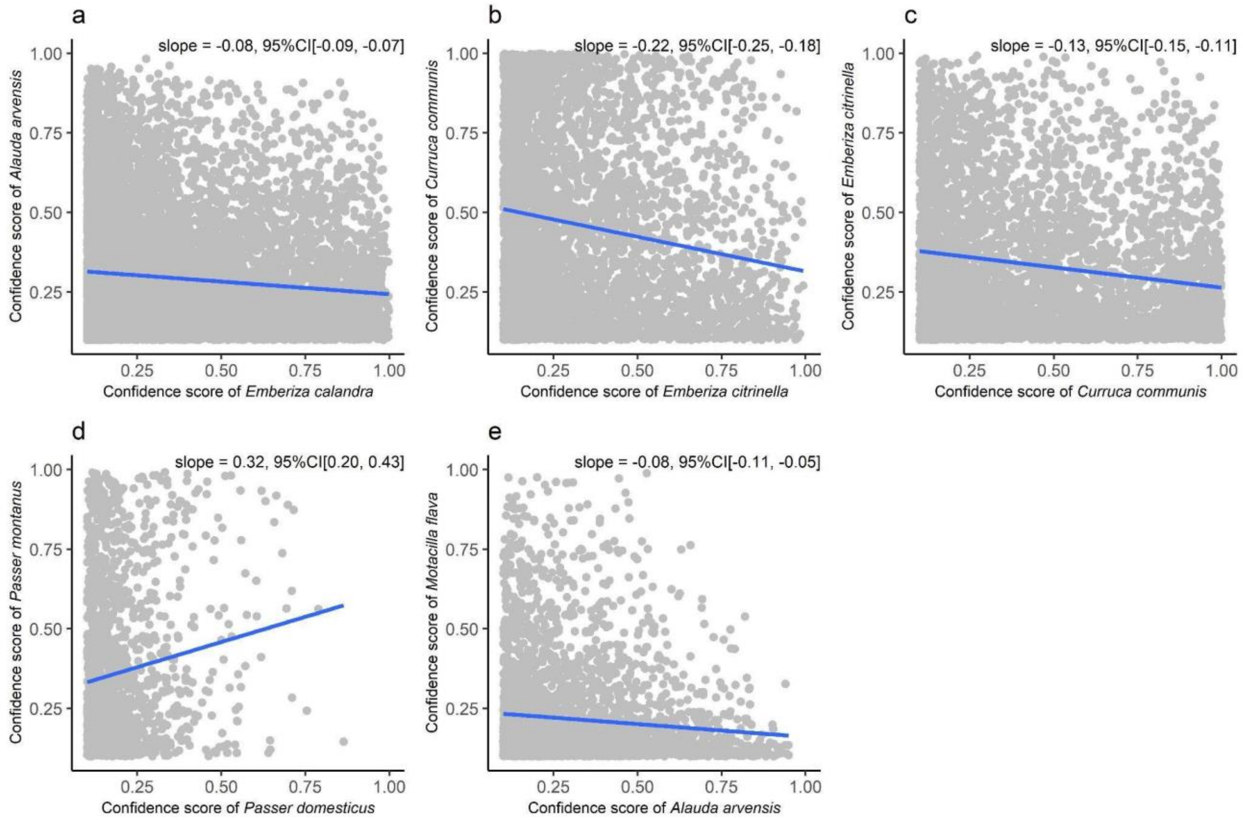

Figure S9. Relationships of confidence scores between five target species and the most commonly co-identified species at the same timing of 3-second BirdNET identification. x-&y-axes represent, respectively, co-identification of non-target and target species. On the upper right of each panel, slope and its 95% confidence interval are shown. **a**. Eurasian skylark (*Alauda arvensis*). **b**. common whitethroat (*Curruca communis*), **c**. yellowhammer (*Emberiza citrinella*), **d**. Eurasian tree sparrow (*Passer montanus*), and **e**. Western yellow wagtail (*Motacilla flava*).

REFERENCES

Chambert, T., B. R. Hossack, L. Fishback, and J. M. Davenport. 2016. "Estimating Abundance in the Presence of Species Uncertainty." *Methods in Ecology and Evolution* 7: 1041-1049. https://doi.org/10.1111/2041-210X.12570.

Chambert, T., E. H. C. Grant, D. A. Miller, J. D. Nichols, K. P. Mulder, and A. B. Brand. 2018. "Two-Species Occupancy Modelling Accounting for Species Misidentification and Non-detection." *Methods in Ecology and Evolution* 9: 1468-1477. https://doi.org/10.1111/2041-210X.12985.

Cole, J. S., N. L. Michel, S. A. Emerson, and R. B. Siegel. 2022. "Automated Bird Sound Classifications of Long-Duration Recordings Produce Occupancy Model Outputs Similar to Manually Annotated Data." *Ornithological Applications* 124: 1–15. https://doi.org/10.1093/ornithapp/duac003.

Darras, K. F. A., N. Pérez, L. Dilong, T. Hanf-Dressler, M. Markolf, T. C. Wanger, and A. F. Cord. 2024. "ecoSound-Web: An Open-Source, Online Platform for Ecoacoustics." *F1000Research* 9:1224. https://f1000research.com/articles/9-1224/v3.

Darras, K. F. A., P. Pütz, Fahrurrozi, K. Rembold, and T. Tscharntke. 2016. "Measuring Sound Detection Spaces for Acoustic Animal Sampling and Monitoring." *Biological Conservation* 201: 29–37. https://doi.org/10.1016/j.biocon.2016.06.021.

European Environment Agency. 2023. "Small Woody Features 2018 (Raster 5 m), Europe, 3-Yearly, May 2023." EEA Geospatial Data Catalogue. 2023. https://sdi.eea.europa.eu/catalogue/srv/api/records/a8e683b1-2f96-45c8-827f-580a79413018.

Hersbach, H., B. Bell, P. Berrisford, G. Biavati, A. Horányi, J. Muñoz Sabater, J. Nicolas, C. Peubey, R. Radu, I. Rozum, D. Schepers, A. Simmons, C. Soci, D. Dee, J. N. Thépaut. 2023. "ERA5 Hourly Data on Pressure Levels from 1940 to Present. Copernicus Climate Change Service (C3S) Climate Data Store (CDS)." August 16, 2023. https://doi.org/10.24381/cds.bd0915c6.

Hijmans, R. J., S. Phillips, J. Leathwick, and J. Elith. 2021. "dismo: Species Distribution Modeling. R Package Version 1.3-5." https://CRAN.R-Project.Org/Package=dismo.

Hines, J. E., J. D. Nichols, J. A. Royle, D. I. Mackenzie, A. M. Gopalaswamy, N. S. Kumar, and K. U. Karanth. 2010. "Tigers on Trails: Occupancy Modeling for Cluster Sampling." *Ecological Applications* 20: 1456–66. https://doi.org/10.1890/09-0321.1.

Knight, E. C., and E. M. Bayne. 2019. "Classification Threshold and Training Data Affect the Quality and Utility of Focal Species Data Processed with Automated Audio-Recognition Software." *Bioacoustics* 28: 539–54. https://doi.org/10.1080/09524622.2018.1503971.

Malinowski, R., S. Lewiński, M. Rybicki, E. Gromny, M. Jenerowicz, M. Krupiński, A. Nowakowski, C. Wojtkowski, M. Krupiński, E. Krätzschmar, P. Schauer. 2020. "Automated Production of a Land Cover/Use Map of Europe Based on Sentinel-2 Imagery." *Remote Sensing* 12: 3523. https://doi.org/10.3390/rs12213523.

Nichols, J. D., L. L. Bailey, A. F. O'Connell Jr., N. W. Talancy, E. H. C. Grant, A. T. Gilbert, E. M. Annand, T. P. Husband, and J. E. Hines. 2008. "Multi-Scale Occupancy Estimation and Modelling Using Multiple Detection Methods." *Journal of Applied Ecology* 45: 1321–29. https://doi.org/10.1111/j.1365-2664.2008.01509.x.

Rhinehart, T. A., D. Turek, and J. Kitzes. 2022. "A Continuous-Score Occupancy Model That Incorporates Uncertain Machine Learning Output from Autonomous Biodiversity

Surveys." *Methods in Ecology and Evolution* 13: 1778–89. https://doi.org/10.1111/2041-210X.13905.

Royle, J. A., and M. Kéry. 2007. "A Bayesian State-Space Formulation of Dynamic Occupancy Models." *Ecology* 88: 1813–23. https://doi.org/10.1890/06-0669.1.

Rushing, C. S., J. A. Royle, D. J. Ziolkowski, and K. L. Pardieck. 2019. "Modeling Spatially and Temporally Complex Range Dynamics When Detection Is Imperfect." *Scientific Reports* 9: 1–9. https://doi.org/10.1038/s41598-019-48851-5.

Saas, Y., and F. Gosselin. 2014. "Comparison of Regression Methods for Spatially-Autocorrelated Count Data on Regularly- and Irregularly-Spaced Locations." *Ecography* 37: 476–89. https://doi.org/10.1111/j.1600-0587.2013.00279.x.

Somervuo, P., P. Lauha, and T. Lokki. 2023. "Effects of Landscape and Distance in Automatic Audio Based Bird Species Identification." *The Journal of the Acoustical Society of America* 154: 245–54. https://doi.org/10.1121/10.0020153.

Tanaka, R. 1980. "Controversial Problems in Advanced Research on Estimating Population Densities of Small Rodents." *Researches on Population Ecology* 22: 1–67. https://doi.org/10.1007/BF02530858.

Zhang, H. T., W. Y. Guo, and W. T. Wang. 2023. "The Dimensionality Reductions of Environmental Variables Have a Significant Effect on the Performance of Species Distribution Models." *Ecology and Evolution* 13: e10747. https://doi.org/10.1002/ece3.10747.
