# Supplementary material for: A classification‐occupancy model based on automatically identified species data
Source: Ecology. 2025 May 7;106(5):e70086. doi: 10.1002/ecy.70086 (PMC12056683; doi:10.1002/ecy.70086)
Supplement: Supplementary file 2 — Appendix S2. [file ECY-106-e70086-s002.pdf]

Appendix S2

Journal: Ecology

Title: A classification-occupancy model based on automatically identified species data

Authors: Ryo Ogawa, Frédéric Gosselin, Kevin F. A. Darras, Stephanie Roilo, and Anna F. Cord

AIM

This guide provides a straightforward approach to run a classification-occupancy model using BirdNET-based skylark detection data. It is designed for;
1) an easy and quick practical introduction to the model;
2) diagnose the model by visual inspection of traceplot and goodness-of-fit test; and
3) applied users who want to develop this model further.

For a deeper understanding of the modeling framework, please refer to our main paper.

BEFORE START

To follow this tutorial, please ensure that you have downloaded the necessary software and data before starting this tutorial.

- JAGS software (Plummer 2003). This software is designed for Bayesian statistics. While we aim to make it user-friendly and avoid delving too deep into the complexities of Bayesian statistics, understanding certain components (like Rhat) may require some basic knowledge. This link also provides step-by-step guidance of installation for R, R Studio, JAGS, and rjags (Liu and Abeyratne 2019).

- In cls_occ zip file of the depository, please go to **cls_occ/tutorial/cls_occ_tutorial.RData**. The RData contains species data, environment data, BUGS language, and R functions (if you use your own data, please preprocess your data to this format by yourself).

- R version 4.4.0 or higher

Required packages

In this tutorial, we need “rjags”, “mcmcplots”, “runMCMCbtadjust”, “ggplot2” packages in R (Plummer 2016; Curtis et al. 2018; Gosselin 2024; Hadley 2016).

```
library(rjags)
library(runMCMCbtadjust)
library(mcmcplots)
library(ggplot2)
```

load input data

First, we load `cls_occ_tutorial.RData`.

```
load("cls_occ_tutorial.RData")
```

Input data structure

The Rdata file contains three datasets: `skylark`, `Xocc`, and `Xdet`. To run the classification-occupancy model with your own data, it is necessary to preprocess your data to match this structure. Therefore, we initially explain the data structure.

Skylark detection data

```
head(skylark)

##            birdID siteID           occID  score
## 1 Alauda arvensis    A04 20220523_053000 0.3981
## 2 Alauda arvensis    A04 20220523_063000 0.4774
## 3 Alauda arvensis    A04 20220523_073000 0.2370
## 4 Alauda arvensis    A04 20220523_093000 0.3467
## 5 Alauda arvensis    A04 20220524_073000 0.1050
## 6 Alauda arvensis    A04 20220525_053000 0.5221
```

This table consists of four columns:

- `birdID`: ID of bird species (here only Eurasian skylark, *Alauda arvensis*, is listed).
- `siteID`: ID of each survey site.
- `occID`: ID of each survey event at a site, formatted as YYYYMMDD_HHMMSS as the starting time of recording.
- `score`: A confidence score ranging from 0.1 to 1.0 (followed by default setting of BirdNET Analyzer). This represents the highest BirdNET confidence scores for each site and event.

Occupancy covariate data

```
head(Xocc)

##   siteID int        PC1       PC2        PC3
## 1    A04   1 -2.4292983 -2.492559 -0.6075544
## 2    A06   1 -2.6662030  2.765582  0.5762272
## 3    A08   1  2.3070278 -1.263063  1.0401427
## 4    A10   1 -1.5807589  1.141106 -0.2368587
## 5    A12   1 -0.9109296  1.156715  0.5825078
## 6    A16   1  1.6489820  1.123012 -1.0104176
```

The occupancy covariate data has five columns: `siteID`, intercept (`int`), and the first three principal components (`PC1`, `PC2`, and `PC3`) used as predictors in our analyses. Note this data is at the site level.

Detection covariate data

```
head(Xdet)

##   siteID           occID int     swind
## 1    A04 20220523_053000   1 0.1573703
## 2    A04 20220523_063000   1 0.1834594
## 3    A04 20220523_073000   1 0.4575492
## 4    A04 20220523_083000   1 0.6387390
## 5    A04 20220523_093000   1 0.8395402
## 6    A04 20220523_103000   1 1.0332101
```

The detection covariate data consists of four columns: `siteID`, `occID`, intercept (`int`), and the wind speed (`swind`) that has been log-transformed and scaled. Note this data is at the site-by-occasion level.

JAGS code

The code of the classification-occupancy model (i.e., "FN+FP" model in the main text) is also saved in `cls_occ_tutorial.RData`. You also examine the model structure by returning `cls_occ_model`. The JAGS code is also available in the repository file `.../cls_occ/jagscodes/model4.txt`

```
cat(cls_occ_model)
```

Note that true and false positive detection error probabilities, respectively denoted as *p* and *q* in the main text and Appendix S1: Table S1, are referred to as *tp* and *fp* in the BUGS language code. The JAGS codes of "Basic", "FN", and "FP" models are available in the repository file `.../cls_occ/jagscodes/model1.txt`, `.../cls_occ/jagscodes/model2.txt`, and `.../cls_occ/jagscodes/model3.txt`, respectively.

QUICK GUIDE

Compiling input data for classification-occupancy modelling framework

We created the "quick_compile()" function, saved in `cls_occ_tutorial.RData`, to prepare all necessary input data for the classification-occupancy model. The argument `i_model` indicates a model type: "Basic" (`i_model = 1`), "FN" (`i_model = 2`), "FP" (`i_model = 3`), or "FN+FP" (`i_model = 4`). Here, we compile input data for the "FN+FP" model;

```
compiled <- quick_compile(sp_d = skylark,       # Species detection data
                          Xocc = Xocc,          # Occupancy covariate data
                          Xdet = Xdet,          # Detection covariate data
                          i_model = 4)          # model type (i.e., “FN+FP”)
```

Parameters to be saved

Before running the model, we need to define the parameters for monitoring and set the JAGS specifications. We make two vectors: 1) parameters to be monitored for parameter convergence (`params.conv`); and 2) parameters to be saved for variable inferences and model fitness (`params.save`);

```
params.conv <- c("mu", "sigma", "u.b", "v.b", "fp")      # Priors
params.save <- c("mu", "sigma", "u.b", "v.b", "fp",      # Priors
                 "mean.fp",                              # Derived quantity
                 "chifit.actual", "chifit.sim",          # GoF
                 "ftfit.actual", "ftfit.sim",            # GoF
                 "sosfit.actual", "sosfit.sim")          # GoF
```

Run model with runMCMC_btadjust

In Bayesian modeling, we predefine parameters like adaptations, chains, thinning, burn-in, and iterations. Although crucial, their setup for achieving MCMC convergence while maintaining adequate sample size is complex. Fortunately, the R package "runMCMCbtadjust" automates this process. Here, We set a minimum of 1,000 effective values and a maximum Rhat of 1.1 over monitored parameters. **Be aware that it will take a long time to reach successful parameter convergence with sufficient effective sample size (i.e., burn-ins = 5,000, thinning rates = 666, and posterior samples = 1,672,637 in skylark data; Appendix S1: Table S5).**

```
nc = 3 # Number of chain
na = 1000;ni = 10000;nt = 1;nb = 5000 # minimum value setting of adaptation,
posterior sampling, thinning rate, and burn-in.
out<-runMCMC_btadjust(MCMC_language="Jags",
                      code=cls_occ_model,
                      data=compiled$bdata,
                      Nchains=nc,
                      inits=lapply(1:nc,function(x){compiled$inits()}),
                      params.conv=params.conv,
                      params.save=params.save,
                      nburnin.min=nb,
                      nburnin.max=Inf,
                      thin.min=nt,
                      thin.max=1000,  # could be Inf
                      niter.min=ni,
                      niter.max=Inf,
                      neff.min=1000,
                      conv.max=1.1,
                      control=list(time.max=50000, # maximum time (in sec)
                                   print.diagnostics=TRUE),
                      control.MCMC=list(n.adapt=na, parallelize=T))
```

Check parameter convergence by traceplot

We visually ensure that all parameters being monitored have converged by traceplot. plot(out) will also work, but given the amount of priors in *fp*, running plot(out) may freeze the Plots window in RStudio. Therefore, we will use the traplot() function in the "mcmcplots" package in R.

```
traplot(out, parms = c("mu", "sigma", "u.b","v.b"))
```

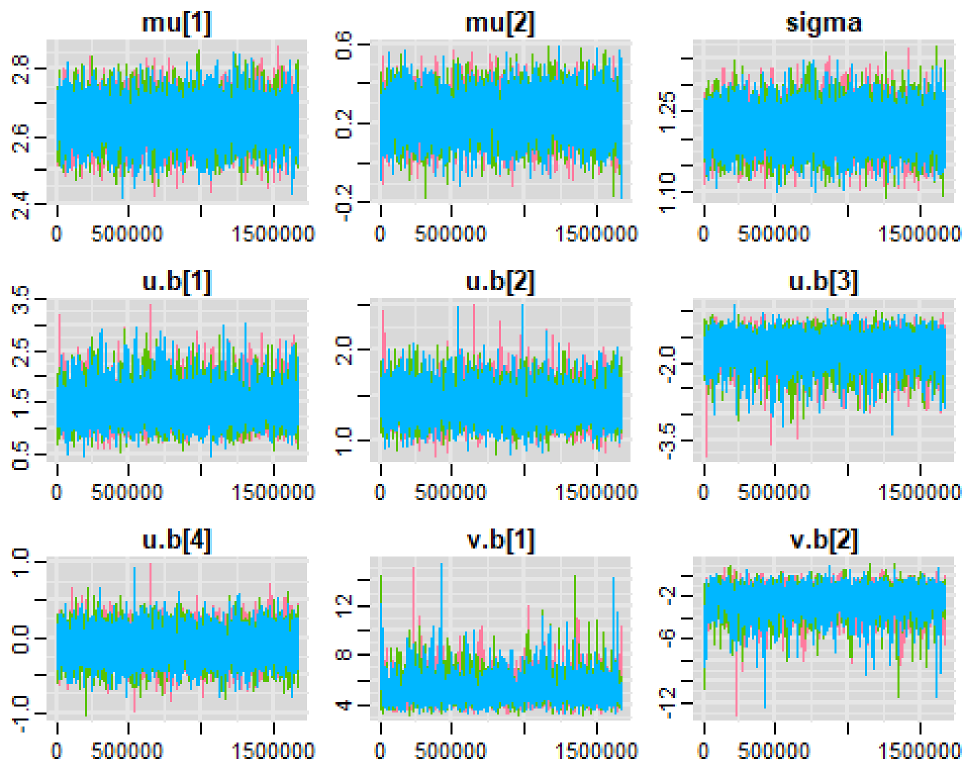


```
### We have to check all false positive error priors, but here we will reduce them due to the amount of traceplots to be inspected.
# fps <- paste0("fp[",1:49,",1]")
# traplot(out, parms = fps)
# fps <- paste0("fp[",1:49,",2]")
# traplot(out, parms = fps)
# fps <- paste0("fp[",1:49,",3]")
# ...
```

Goodness-of-Fit test

Next, we need to check the goodness-of-fit (GoF) test: 1) chi-square discrepancy measures for the occupancy model and 2) sum of residual discrepancy measures for the classification model. For now, we regard the model fitting as acceptable if the posterior predictive p-values (PPP) lie between 0.05 and 0.95;

```
out2 <- do.call("rbind", out)
chifit_ppp <- mean(out2[,"chifit.actual"]<out2[,"chifit.sim"])
sosfit_ppp <- mean(out2[,"sosfit.actual"]<out2[,"sosfit.sim"])
paste0("PPP of Chi-square discrepancy measure: ", round(chifit_ppp,3))

## [1] "PPP of Chi-square discrepancy measure: 0.376"

paste0("PPP of sum-of-square discrepancy measure: ", round(sosfit_ppp,3))

## [1] "PPP of sum-of-square discrepancy measure: 0.515"
```

Visual investigation of posterior predictive check is as follows;

```
ggplot(out2, aes(x=chifit.actual,y=chifit.sim)) +
    geom_point() +
    xlab("Observed chi-square residual") +
    ylab("Simulated chi-square residual") +
    geom_abline(intercept = 0, slope = 1) +
    theme_classic()
```

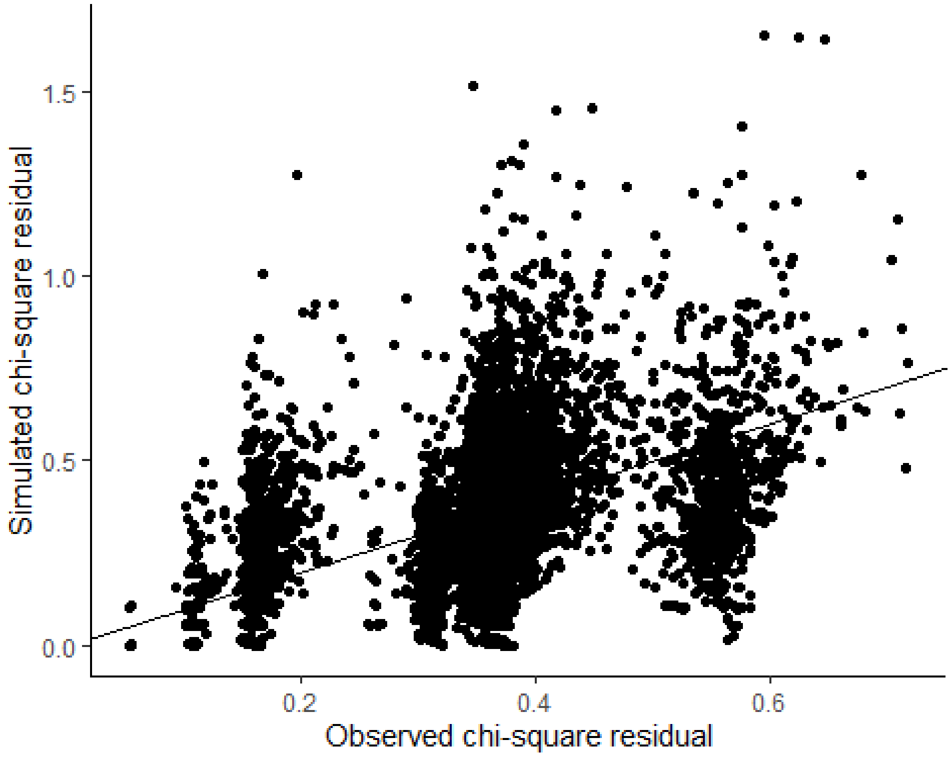


```
ggplot(out2, aes(x=sosfit.actual,y=sosfit.sim)) +
    geom_point() +
    xlab("Observed sum of squared residual") +
    ylab("Simulated sum of squared residual") +
    geom_abline(intercept = 0, slope = 1) +
    theme_classic()
```

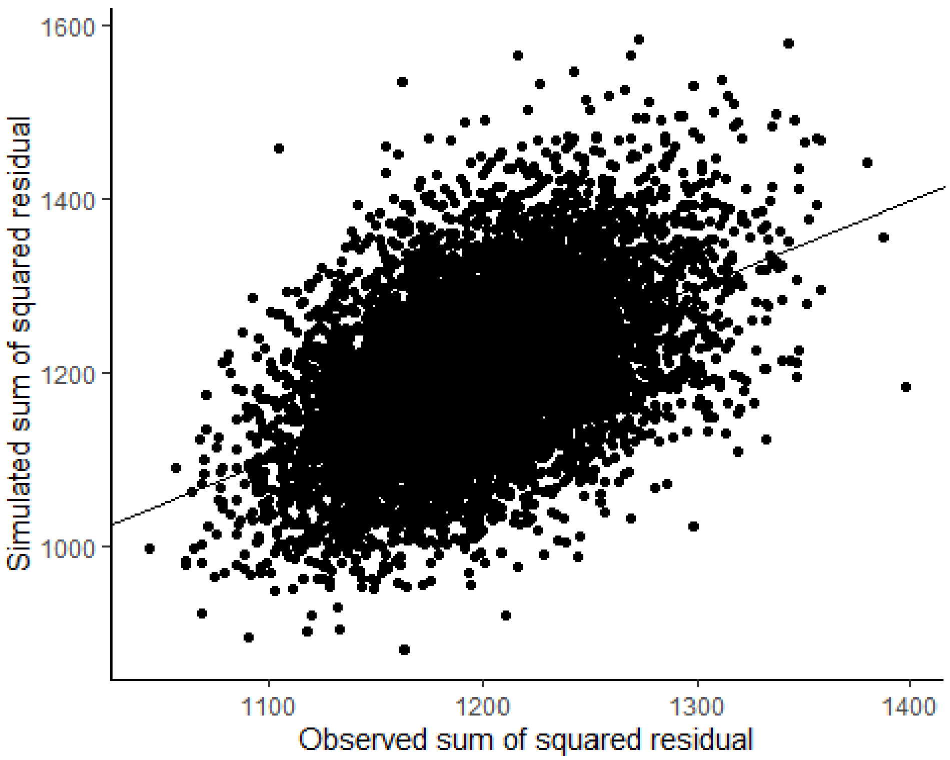

As we discussed in the main text, GoF test needs further exploration for occupancy models.

Output summary

You can check summary output of all monitored parameters;

```
s <- summary(out)
g <- grep(c("mu|sigma|u.b|v.b"), rownames(s$statistics))
s$quantiles[g,]

##              2.5%        25%        50%        75%      97.5%
## mu[1]    2.5191305  2.6047178  2.6459866  2.6871974  2.7681476
## mu[2]    0.0328056  0.1673371  0.2392979  0.3106542  0.4433246
## sigma    1.1442692  1.1925296  1.2183676  1.2438124  1.2956951
## u.b[1]   0.8788071  1.2095960  1.4104509  1.6365533  2.2247035
## u.b[2]   1.0715281  1.2663227  1.3871013  1.5152891  1.8028878
## u.b[3] -2.4370108 -1.8767253 -1.6786119 -1.5103028 -1.2374854
## u.b[4] -0.5168696 -0.2517504 -0.1123444  0.0302281  0.3123824
## v.b[1]   3.7578613  4.5199145  5.0472314  5.7289912  8.0438388
## v.b[2] -5.5761848 -3.0610870 -2.1904863 -1.4454190 -0.3239927

# s$statistics[g,] # this is also informative
```

These 9 rows are the monitored parameters from the classification-occupancy model;

- `mu[1]`: location parameter (mean) of true confidence scores (logit-transformed)
- `mu[2]`: location parameter (mean) of false confidence scores (logit-transformed)

- `sigma`: scale parameter (standard deviation) of true and false confidence scores (logit-transformed)
- `u.b[1-4]`: estimates of occupancy coefficients (`u.b[1]`=intercept and `u.b[2-4]`=PC1-3)
- `v.b[1-2]`: estimates of detection coefficients (`v.b[1]`=intercept and `v.b[2]`=wind speed)

Users can create a predicted species occupancy map using `u.b` values. Please note that the specifics of spatial mapping are not covered in this tutorial.

DETAIL FOR COMPILED DATA

If you prepare for input data like the aforementioned example, JAGS will start running the model without errors. We believe that this base model can be adapted to various occupancy models, including dynamic occupancy models (Royle and Kéry 2007), multi-species occupancy models (Tobler et al. 2019), or occupancy models accounting for spatial/temporal autocorrelations (Rushing et al. 2019). Therefore, you may be interested in what quick_compile() has returned as output for your future research. We will detail the data structure in this section.

```
str(compiled$bdata)
```

```
## List of 13
##  $ y       : num [1:49, 1:18] 1 1 1 1 1 1 0 1 0 1 ...
##   ..- attr(*, "dimnames")=List of 2
##   .. ..$ site    : chr [1:49] "A04" "A06" "A08" "A10" ...
##   .. ..$ occasion: chr [1:18] "20220523_053000" "20220523_063000"
"20220523_073000" "20220523_083000" ...
##  $ w       : num [1:49, 1:18] NA NA NA NA NA NA 0 NA 0 NA ...
##   ..- attr(*, "dimnames")=List of 2
##   .. ..$ site    : chr [1:49] "A04" "A06" "A08" "A10" ...
##   .. ..$ occasion: chr [1:18] "20220523_053000" "20220523_063000"
"20220523_073000" "20220523_083000" ...
##  $ siteid  : int [1:808] 1 1 1 1 1 1 1 1 1 2 ...
##  $ occid   : int [1:808] 1 2 3 5 9 13 14 15 17 1 ...
##  $ score   : num [1:808] -0.4134 -0.0905 -1.1692 -0.6336 -2.1429 ...
##  $ nsites  : int 49
##  $ noccs   : int 18
##  $ nsamples: int 808
##  $ Xocc    :'data.frame':    49 obs. of  4 variables:
##   ..$ int: num [1:49] 1 1 1 1 1 1 1 1 1 1 ...
##   ..$ PC1: num [1:49] -2.429 -2.666 2.307 -1.581 -0.911 ...
##   ..$ PC2: num [1:49] -2.49 2.77 -1.26 1.14 1.16 ...
##   ..$ PC3: num [1:49] 0.608 -0.576 -1.04 0.237 -0.583 ...
##  $ Xdet    : num [1:49, 1:18, 1:2] 1 1 1 1 1 1 1 1 1 1 ...
##   ..- attr(*, "dimnames")=List of 3
##   .. ..$ site    : chr [1:49] "A04" "A06" "A08" "A10" ...
##   .. ..$ occasion: chr [1:18] "20220523_053000" "20220523_063000"
"20220523_073000" "20220523_083000" ...
```

```
##   .. ..$ variable: chr [1:2] "int" "swind"
##  $ Vocc    : int 4
##  $ Vdet    : int 2
##  $ e       : num 0.001
```

The matrix y represents encounter history, with 1 for detection and 0 for non-detection. The row names are site IDs, and column names are event (or occasion) IDs. The structure of the encounter history data is detailed below:

```
compiled$bdata$y[1:10, 1:4]
```

```
##      occasion
## site  20220523_053000 20220523_063000 20220523_073000 20220523_083000
##   A04               1               1               1               0
##   A06               1               1               1               1
##   A08               1               1               1               1
##   A10               1               1               1               1
##   A12               1               1               1               1
##   A16               1               1               1               1
##   A17               0               0               0               0
##   A19               1               1               1               1
##   A23               0               0               0               0
##   A28               1               0               0               0
```

The `w` matrix (binary process of detection truth; i.e., true=0 and false=1) has the same structure as `y`. However, in `w`, the 1s from `y` are replaced with `NA`.

```
compiled$bdata$w[1:10, 1:4]
```

```
##      occasion
## site  20220523_053000 20220523_063000 20220523_073000 20220523_083000
##   A04              NA              NA              NA               0
##   A06              NA              NA              NA              NA
##   A08              NA              NA              NA              NA
##   A10              NA              NA              NA              NA
##   A12              NA              NA              NA              NA
##   A16              NA              NA              NA              NA
##   A17               0               0               0               0
##   A19              NA              NA              NA              NA
##   A23               0               0               0               0
##   A28              NA               0               0               0
```

In the classification model, each BirdNET confidence score (808 in total; `nsamples`) requires a specified `siteID` and `occID`. These need to be integers for JAGS: `siteid` for site ID and `occid` for event ID.

The `score` vector consists of logit-transformed values derived from BirdNET confidence scores. Users may change the type of transformation, depending on the range of confidence scores.

The values `nsites` and `nocc` represent the number of sampling sites and events, respectively. `Xocc` maintains the same data structure as the original input data.

The data structure of `Xdet` has been transformed from a matrix to a 3D-array, encompassing site, event, and variable name at each dimension.

```
compiled$bdata$Xdet[1:10, 1:4, ]

## , , variable = int
##
##      occasion
## site  20220523_053000 20220523_063000 20220523_073000 20220523_083000
##   A04               1               1               1               1
##   A06               1               1               1               1
##   A08               1               1               1               1
##   A10               1               1               1               1
##   A12               1               1               1               1
##   A16               1               1               1               1
##   A17               1               1               1               1
##   A19               1               1               1               1
##   A23               1               1               1               1
##   A28               1               1               1               1
##
## , , variable = swind
##
##      occasion
## site  20220523_053000 20220523_063000 20220523_073000 20220523_083000
##   A04      0.15737029       0.1834594       0.4575492       0.6387390
##   A06      0.15737029       0.1834594       0.4575492       0.6387390
##   A08      0.06971582       0.1534910       0.4755518       0.5548227
##   A10      0.06971582       0.1534910       0.4755518       0.5548227
##   A12      0.06971582       0.1534910       0.4755518       0.5548227
##   A16      0.15737029       0.1834594       0.4575492       0.6387390
##   A17      0.06971582       0.1534910       0.4755518       0.5548227
##   A19      0.15737029       0.1834594       0.4575492       0.6387390
##   A23      0.15737029       0.1834594       0.4575492       0.6387390
##   A28      0.06971582       0.1534910       0.4755518       0.5548227
```

REFERENCES

Curtis, S. McKay. 2018. "mcmcplots: Create Plots from MCMC Output." R package version 0.4.3. https://cran.r-project.org/web/packages/mcmcplots/index.html.

Gosselin, F.. 2024. "runMCMCbtadjust: Runs Monte Carlo Markov Chain - With Either 'JAGS,' 'Nimble' or 'Greta' - While Adjusting Burn-in and Thinning Parameters." R package version 1.1.0. https://cran.r-project.org/web/packages/runMCMCbtadjust/index.html.

Hadley, W.. 2016. *ggplot2: Elegrant Graphics for Data Analysis*. Springer.

Liu, Y., and A. I. Abeyratne. 2019. "Appendix A Guidance for Installing R, R Studio, JAGS, and Rjags." In *Practical Applications of Bayesian Reliability*, 279–80. John Wiley & Sons, Ltd. https://onlinelibrary.wiley.com/doi/abs/10.1002/9781119287995.app1.

Plummer, M.. 2003. “JAGS: A Program for Analysis of Bayesian Graphical Models Using Gibbs Sampling.” In *Proceedings of the 3rd International Workshop on Distributed Statistical Computing (DSC 2003)*, 1–10.

Plummer, M.. 2016. “rjags: Bayesian Graphical Models Using MCMC.” R package version 4-6. https://cran.r-project.org/package=rjags.

Royle, J. A., and M. Kéry. 2007. “A Bayesian State-Space Formulation of Dynamic Occupancy Models.” *Ecology* 88: 1813–23. https://doi.org/10.1890/06-0669.1.

Rushing, C. S., J. A. Royle, D. J. Ziolkowski, and K. L. Pardieck. 2019. “Modeling Spatially and Temporally Complex Range Dynamics When Detection Is Imperfect.” *Scientific Reports* 9: 1–9. https://doi.org/10.1038/s41598-019-48851-5.

Tobler, M. W., M. Kéry, F. K. C. Hui, G. Guillera-Arroita, P. Knaus, and T. Sattler. 2019. “Joint Species Distribution Models with Species Correlations and Imperfect Detection.” *Ecology* 100: 1–14. https://doi.org/10.1002/ecy.2754.
